# Machine learning-based clustering to identify the combined effect of the DNA fragmentation index and conventional semen parameters on in vitro fertilization outcomes

**DOI:** 10.1186/s12958-023-01080-y

**Published:** 2023-03-15

**Authors:** Tianwen Peng, Chen Liao, Xin Ye, Zhicong Chen, Xiaomin Li, Yu Lan, Xin Fu, Geng An

**Affiliations:** Department of Obstetrics and Gynecology, Center of Reproductive Medicine, Key Laboratory for Major Obstetric Diseases of Guangdong Province, The Third Affiliated Hospital of Guangzhou Medical University, Guangzhou, 510150 Guangdong China

**Keywords:** Sperm DNA fragmentation index, Routine semen parameters, Coexposure, K-means clustering, IVF outcomes

## Abstract

**Background:**

Previous studies have demonstrated an association between male sperm quality and assisted reproduction outcomes, focusing on the effects of individual parameters and reaching controversial conclusions. The WHO 6th edition manual highlights a new semen assay, the sperm DNA fragmentation index, for use after routine semen examination. However, the combined effect of the sperm DNA fragmentation index (DFI) and routine semen parameters remains largely unknown.

**Methods:**

We assessed the combined effect of the sperm DFI and conventional semen parameters on single fresh conventional IVF outcomes for infertile couples from January 1, 2017, to December 31, 2020. IVF outcomes were obtained from the cohort database follow-up records of the Clinical Reproductive Medicine Management System of the Third Affiliated Hospital of Guangzhou Medical University. An unsupervised K-means clustering method was applied to classify participants into several coexposure pattern groups. A multivariate logistic regression model was used for statistical analysis.

**Results:**

A total of 549 live births among 1258 couples occurred during the follow-up period. A linear exposure–response relationship was observed among the sperm DFI, sperm motility, and IVF outcomes. In multivariable adjustment, increased sperm DFI values and decreased sperm motility and semen concentration levels were associated with reduced odds of favourable IVF outcomes. Four coexposure patterns were generated based on the sperm DFI and the studied semen parameters, as follows: Cluster 1 (low sperm DFI values and high sperm motility and semen concentration levels), Cluster 2 (low sperm DFI values and moderate sperm motility and semen concentration levels), Cluster 3 (low sperm DFI values and low sperm motility and semen concentration levels) and Cluster 4 (high sperm DFI values and low sperm motility and semen concentration levels). Compared with those in Cluster 1, participants in Cluster 3 and Cluster 4 had lower odds of a live birth outcome, with odds ratios (95% confidence intervals [CIs]) of 0.733 (0.537, 0.998) and 0.620 (0.394, 0.967), respectively.

**Conclusions:**

When combined with low sperm DFI values, there was no significant difference between high or moderate sperm concentration and motility levels, and both were associated with favourable IVF outcomes. Low sperm parameter levels, even when DFI values remain low, may still lead to poor IVF outcomes. Participants with high sperm DFI values and low sperm motility and semen concentration levels had the worst outcomes. Our findings offer a novel perspective for exploring the joint effects of sperm DFI and routine semen parameter values.

**Supplementary Information:**

The online version contains supplementary material available at 10.1186/s12958-023-01080-y.

## Background

Male factor infertility is one of the indications for couples to consider conventional in vitro fertilization (IVF) to bear offspring [1–3]. Semen analysis remains the most common method to assess male infertility [4]. However, nearly 15% of infertile men have normal semen parameters [5]. Sperm concentration and motility have limited predictive value for conventional IVF outcomes [6–9]. This implies that subcellular or nuclear factors ignored by routine semen analysis may contribute to male factor infertility and affect the outcomes of IVF. Adequate assessment of male sperm quality is essential to reduce patient burden and improve IVF outcomes [10–12].

Sperm DNA integrity, which is necessary for the accurate transmission of paternal genetic information [5], has become one of the most discussed and promising biomarkers of male infertility. Animal studies have shown that after the union of paternal and maternal DNA, DNA damage from the paternal source is examined and repaired in fertilized eggs, and the failure of this results in embryonic death or affects the ability of the embryo to continue to develop [13, 14]. In 2021, the WHO published the Laboratory Manual for the Examination and Processing of Human Semen (6th edition), highlighting a new semen test, the sperm DNA fragmentation index [5].

Numerous clinical studies have been conducted to elucidate any correlation between sperm DNA integrity and IVF outcomes, but the findings remain partially controversial. Some studies suggest that sperm DNA damage has a detrimental effect on clinical pregnancy and live birth outcomes after conventional IVF [15–19]. Others have not identified a connection between sperm DNA integrity and IVF outcomes [20–22]. One potential reason is that the effect on certain semen parameters (e.g., sperm concentration and motility) [6–9] or sperm DNA integrity alone is frequently studied, but few studies have focused on the combined effect of sperm DNA integrity and semen parameters on IVF outcomes. Other factors, such as not excluding interference from multiple cycles and cycle types [23], varying patient selection criteria [24–26], not controlling for potential confounders, the lack of standardized methods to detect sperm DNA integrity, and limited study sample sizes, could account for the inconsistency among study results.

In the current study, we mainly aimed to elucidate the joint effects of the sperm DNA fragmentation index (DFI) and routine semen parameter values on the live birth, clinical pregnancy, and positive *β*-hCG outcomes of single fresh conventional IVF cycles. As secondary aims, we investigated the separate impact of the sperm DFI and certain semen parameters on IVF outcomes.

## Materials and methods

### Study population

These data were extracted from a longitudinal cohort of ART cycles between 2017 and 2020 at the Center for Reproductive Medicine, The Third Hospital of Guangzhou Medical University. In retrieving the electronic health data, this study limited ART to conventional IVF and defined the female population as follows: women must be ≤ 38 years old, have a body mass index of 18 kg/m^2^ to 35 kg/m^2^, serum anti-Müllerian hormone (AMH) levels must be ≥ 1.2 ng/ml, and the average follicle counts must be ≥ 5.

In the current analysis, we excluded couples with the following conditions, including failed ejaculation on oocyte retrieval day, failed oocyte retrieval, recurrent abortion, uterine malformations, multiple failed implantations, and the presence of clinically significant chromosomal abnormalities. Additionally, we excluded participants who had no available data on sperm DFI. Non-fresh transplant cycles or fresh cycles that were not the closest to the date of the semen routine and DFI tests were excluded from the study.

### Sperm DNA fragmentation and acridine orange flow cytometry (AO FCM)

The AO FCM was followed as previously described [5, 27–29]. Fresh semen samples after liquefaction were diluted to 1–2 × 10^6^/ml using TNE (10 Tris–Cl, 150 NaCl, and 10 EDTA, pH 7.4). Transfer 200 μl of diluted sample to a flow cytometer tube and add 400 μl of acid detergent (main components are 0.08 M HCl, 150 mM NaCl, 0.1% Triton X-100. pH 1.2) to it. After 30 s, 1.2 ml of a solution containing 0.6 mg/L purified acridine orange (other components include 0.1 M citric acid, 0.2 M Na_2_HPO_4_, 1 mM disodium EDTA and 150 mM NaCl. pH 6.0) was added for staining. The sample was assayed after equilibrating the sample line of the flow cytometer and at least 5000 cells were recorded and counted. Using the properties of AO (green fluorescence for AO bound to double-stranded DNA and red fluorescence for AO bound to single-stranded DNA), data on red and green fluorescence were collected by flow cytometry after excitation with a 488 nm light source. After converting, the ratio of the red fluorescent signal intensity to the sum of the red fluorescent signal intensity and the green fluorescent signal intensity was used to represent the DNA fragmentation index.

### Conventional IVF procedure

The entire IVF procedure generally consists of four stages: controlled ovarian stimulation, oocyte retrieval, embryo transfer, and pregnancy testing [23, 30]. For controlled ovarian stimulation in IVF cycles, three main regimens are used: gonadotropin-releasing hormone (GnRH) antagonist protocol, GnRH agonist protocol, and mild stimulation protocol. GnRH antagonist regimen initiated from day 2 to 3 of the menstrual cycle with 100–300 IU recombinant follicle-stimulating hormone (FSH) (Gonal-F, Merck Serono, S.p.A.) per day. Follicle development was monitored with transvaginal ultrasound. GnRH antagonist was used when a leading follicle reached 12 mm in diameter or since day 5 of ovarian stimulation. GnRH agonist regimen started with triptorelin acetate given at the mid-luteal phase of the previous cycle, followed by 100–300 IU of FSH starting 14 days after downregulation. Patients start mild ovarian stimulation on the 2nd-3rd day of the cycle with recombinant FSH 150 IU/day and clomiphene citrate (CC, 100–150 mg/day) or letrozole (LE, 2.5–5 mg/day). Recombinant hCG (Ovitrelle, Merck Serono) of 250 µg or 6,000–10,000 IU urinary human chorionic gonadotropin (hCG) was injected with the presence of at least two follicles at least 18 mm in diameter or three follicles at least 17 mm in diameter. The oocytes were extracted after 36 h. Luteal phase support with 90 mg vaginal progesterone gel (Crinone, Merck Serono) and 20 mg dydrogesterone (Abbott Biologicals B.V.) was given daily after oocyte retrieval. One to two embryos were transferred 3 or 5 days later based on the embryo stage. A pregnancy test with serum hCG detection was performed 14 days after embryo transfer. If pregnancy was achieved, luteal phase support continued until the 10th week of gestation.

### Outcomes

The primary outcome was live birth after the first fresh transfer. A live birth refers to the delivery of any viable neonate who is 28 weeks or older in gestation [23]. Secondary outcomes include *β*-hCG positive outcomes, clinical pregnancy outcomes, and miscarriage outcomes. A serum human chorionic gonadotropin level of at least 25 mIU/mL at 14 days following embryo transfer was considered to be "*β*-hCG positive". Clinical pregnancy was defined as clinically visible evidence of pregnancy other than biochemical indicators, including an intrauterine gestational sac visible on ultrasound, ectopic pregnancy, miscarriage, or chorionic tissue visible on curettage. Miscarriage was defined as intrauterine pregnancy loss after clinical pregnancy.

### Statistical analysis

Number (percent) was used to describe the category or binary variables, and median (interquartile range, IQR) was used to describe non-normal distribution continuous variables. Correlation coefficients between the studied semen parameters and sperm DFI were calculated using Spearman correlation analysis. We performed Min–Max scaling on the studied semen parameters and sperm DFI to eliminate the effect of magnitude. The "ConsensusClusterPlus" package was used to identify clusters [31]. The procedure was repeated for multiple values of K, and the output was used as a reference to determine the optimal number of clusters. The K-means clustering algorithm, an unsupervised machine learning method, was then used to obtain clustering information based on a determined optimal number of clusters. The Wilcoxon rank sum test was then applied to compare the baseline characteristics of different clusters.

Crude analysis and multivariable logistic regression models were performed to assess the associations between the studied semen parameters, sperm DFI, and IVF outcomes (live birth outcome, clinical pregnancy outcome, and *β*-hCG positive outcome). We computed odds ratios (ORs) and 95% confidence intervals (95% CIs) in the two models. Model 1 was adjusted for the duration of the attempt to conceive, female age, male age, female BMI, and male BMI. Model 2 was additionally adjusted for controlled ovulation stimulation protocols, AMH, E_2_, FSH, endometrial thickness, and the number of oocytes retrieved. Restricted cubic spline functions were employed to portray the relationship between the studied semen parameters/sperm DFI and the outcomes and to calculate the overall and nonlinear *P*-values. We then divided the studied semen parameters and sperm DFI into four quarters, with the lowest category being the reference, to explore their relationship with outcomes separately. To analyze the joint effect of the studied semen parameters and sperm DFI co-exposure on IVF outcomes, we included the identified clusters in the crude analysis and the two multivariable logistic regression models described above. We also used causal mediation analysis (CMA) to explore the mediating effect of fertilization rate on IVF outcomes in different clusters.

The R software, version 4.1.2 (R Project for Statistical Computing), was used for all statistical analyses. A two-sided *P* value < 0.05 was considered statistically significant.

### Ethics

The study protocol was reviewed and approved by the Academic Committee and the Ethics Committee of the Third Affiliated Hospital of Guangzhou Medical University (No.2021229). The need for informed consent was waived by the Ethics Committee due to the retrospective nature of the study. All procedures were carried out following the relevant guidelines and regulations.

## Results

### Baseline characteristics of all patients

**Fig. 1:**
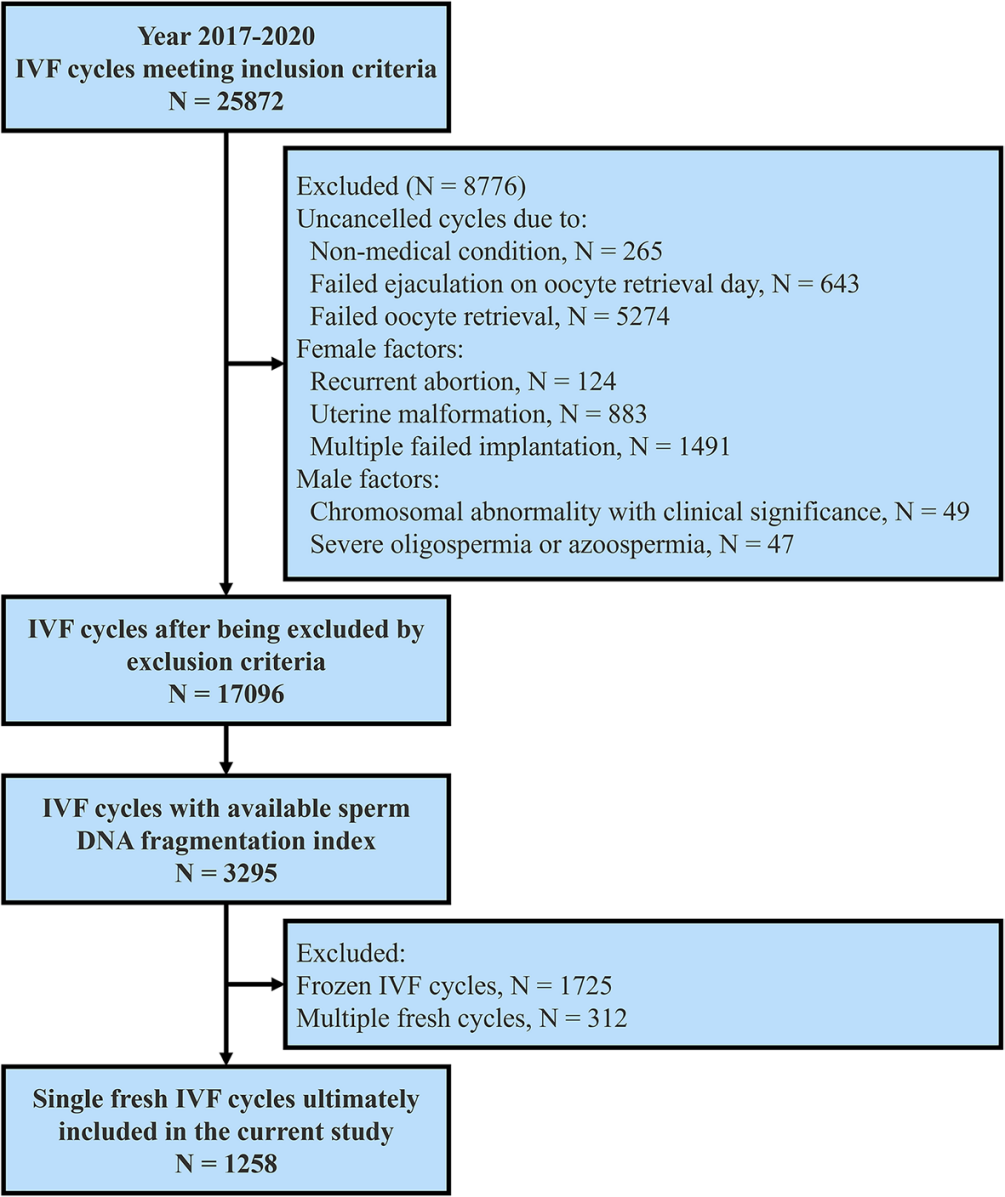
Flow chart for the selection of participants in the cohort study

After all exclusions, a total of 1258 couples undergoing fresh transfer in vitro fertilization cycles were included in the analysis (Fig. 1). In this cohort, 664 (52.8%), 646 (51.4%), and 549 (43.6%) couples had positive *β*-hCG, clinical pregnancy, and live birth outcomes, respectively. The baseline characteristics of all couples in this study are presented in Supplementary Table 1.

The correlation coefficient values between the sperm DNA fragmentation index and the studied routine semen parameters ranged from -0.5 to 0. Correlation coefficient values between two studied routine semen parameters ranged from -0.2 to 0.4. A heatmap showing pairwise correlations among the studied parameters is presented in Supplementary Fig. 1.

**Fig. 2:**
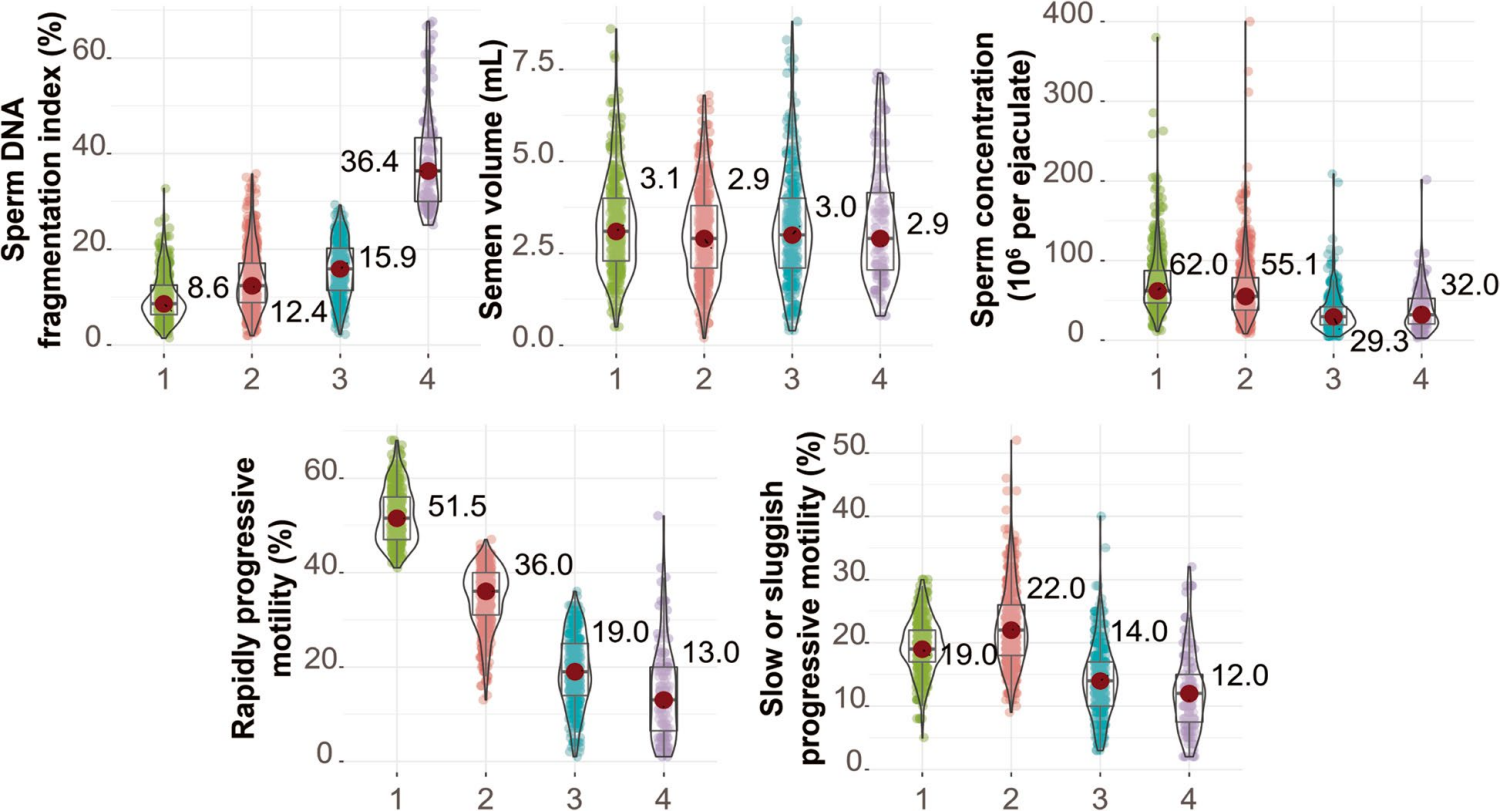
The violin plot of sperm DFI and the studied routine semen parameters stratified by the 4 clusters based on the variables among all participants. Green dots refer to cluster 1 (low-level DFI/high-level semen parameter group); red dots refer to cluster 2 (low-level DFI/median-level semen parameter group); blue dots refer to cluster 3 (low-level DFI/low-level semen parameter); purple dots refer to cluster 4 (high-level DFI/low-level semen parameter)

We considered four as the optimal number of clusters by the cumulative distribution function (CDF) plot (Supplementary Fig. 2(A)), elbow method (Supplementary Fig. 2(B)), consensus matrix heatmap (Supplementary Fig. 2(C)), mean cluster consensus score (Supplementary Fig. 2(D)), and clinical application interpretability. Then, the K-means clustering method was used to cluster all 1258 infertile couples who underwent fresh transfer in vitro fertilization treatment cycles into four clusters. Supplementary Table 2 presents statistics depicting the distributions of the routine semen parameters and sperm DNA fragmentation index values after Min–Max scaling. The clustering results are shown in Supplementary Fig. 3. The violin plot illustrating the features of the four clusters is shown in Fig. 2. Compared with those in the other three clusters, male patients in Cluster 1 had lower median sperm DFI values (8.6% [6.4%, 12.5%]), higher median sperm concentration levels (62.0 [46.9, 87.3] × 10^6^/ml), higher median rapidly progressive motility levels (51.5% [47.0%, 56.0%]), and higher median slow or sluggish progressive motility levels (19.0% [17.0%, 22.0%]). Male patients in Cluster 2 had relatively low median sperm DFI values (12.4% [8.8%, 17.1%]) and intermediate median semen parameter levels. The median sperm DFI value was also relatively low in Cluster 3 (15.9% [11.4%, 20.2%]), while the median semen parameter levels were also low (for example, the median rapidly progressive motility level was 19.0% [14.0%, 25.0%]). Male patients in Cluster 4 had higher median sperm DFI values (36.4% [30.1%, 43.3%]) and lower median semen parameter levels (for example, the median rapidly progressive motility level was 13.0% [6.5%, 20.0%]). Thus, we designated the 'low-level DFI/high-level sperm motility and semen concentration group' as Cluster 1, the 'low-level DFI/median-level sperm motility and semen concentration group' as Cluster 2, the 'low-level DFI/low-level sperm motility and semen concentration group' as Cluster 3, and the 'high-level DFI/low-level sperm motility and semen concentration group' as Cluster 4.

The characteristics of the study participants across the four clusters are shown in Table 1. Compared with those in the other three clusters, the median female age (32.00 years [30.0, 35.0]) and the median male age (36.0 years [31.5, 39.5]) were both higher in Cluster 4 (*P* < 0.05). The duration of the attempt to conceive, male BMI, female BMI, anti-Mullerian hormone level, oestradiol level, follicle-stimulating hormone level, and endometrial thickness on the hCG trigger day were not significantly different among the four clusters. The proportion of participants undergoing controlled ovarian stimulation using the long downregulation protocol was similar in all four clusters (67.7%, 69.7%, 68.1%, and 67.8%, respectively) (*P* = 0.927). Although no significant differences were seen in the number of eggs retrieved among the four clusters, the numbers of fertilized eggs, the numbers of oocytes cleaved, and the numbers of embryos available on Day 3 were lower in Cluster 4 than in the other three clusters in terms of embryo laboratory outcomes. Cluster 4 had the lowest median fertilization rate (*P* < 0.001), but the three clusters had similar median cleavage and D3-available embryo rates.

**Table 1:** Baseline characteristics of 1258 infertile couples clustered in 4 clusters according to sperm DFI and the studied routine semen parameters

**Characteristics**	**Cluster 1**	**Cluster 2**	**Cluster 3**	**Cluster 4**	***P*-value**
N	346	468	329	115	
Duration of attempt to conceive, years	3.0 [2.0, 6.0]	3.0 [2.0, 6.0]	4.0 [2.0, 5.0]	4.0 [2.0, 6.0]	0.521
Male age, years	32.0 [30.0, 35.0]	33.0 [30.0, 36.0]	33.0 [31.0, 37.0]	36.0 [31.5, 39.5]	< 0.001
Female age, years	31.0 [28.0, 33.0]	31.0 [29.0, 34.0]	31.00 [29.0, 34.0]	32.00 [30.0, 35.0]	0.003
Male BMI, kg/m^2^	23.5 [21.2, 25.9]	23.7 [21.7, 26.1]	23.3 [21.3, 25.6]	23.5 [21.3, 26.5]	0.534
Female BMI, kg/m^2^	21.9 [20.0, 23.9]	21.7 [20.0, 23.9]	21.8 [20.3, 23.6]	22.1 [20.8, 23.9]	0.377
Sperm DNA fragmentation index, %	8.6 [6.4, 12.5]	12.4 [8.8, 17.1]	15.9 [11.4, 20.2]	36.4 [30.1, 43.3]	< 0.001
Semen volume, mL	3.1 [2.3, 4.0]	2.9 [2.1, 3.8]	3.0 [2.1, 4.0]	2.9 [2.1, 4.2]	0.182
Sperm concentration, 10^6^ / ml	62.0 [46.9, 87.3]	55.1 [38.1, 78.4]	29.30 [19.3, 41.8]	32.00 [20.6, 52.6]	< 0.001
Rapidly progressive motility, %	51.5 [47.0, 56.0]	36.0 [31.0, 40.0]	19.0 [14.0, 25.0]	13.0 [6.5, 20.0]	< 0.001
Slow or sluggish progressive motility, %	19.0 [17.0, 22.0]	22.0 [18.0, 26.0]	14.0 [10.0, 17.0]	12.0 [7.5, 15.0]	< 0.001
Anti-Mullerian hormone, ng/ml	4.1 [2.6, 6.2]	3.9 [2.4, 6.4]	3.7 [2.6, 6.2]	3.8 [2.6, 5.6]	0.783
Estradiol, pmol/L	117.5 [83.4, 174.3]	126.0 [88.0, 178.0]	125.0 [87.0, 171.0]	121.0 [93.0, 154.5]	0.681
Follicle-stimulating hormone, mIU/ml	5.4 [4.6, 6.1]	5.2 [4.5, 6.1]	5.2 [4.3, 6.3]	5.5 [4.8, 6.3]	0.217
Endometrial thickness on hCG trigger day, mm	10.7 [9.5, 11.6]	10.7 [9.5, 11.8]	10.7 [9.3, 12.0]	10.7 [9.5, 12.0]	0.679
Number of long down-regulation protocol	234 (67.6)	326 (69.7)	224 (68.1)	78 (67.8)	0.927
Number of oocytes retrieved	11.0 [8.0, 14.0]	11.0 [8.0, 14.0]	11.0 [8.0, 14.0]	10.0 [8.0, 14.0]	0.854
Number of fertilized eggs	9.0 [6.0, 11.0]	9.0 [6.0, 12.0]	8.0 [6.0, 11.0]	7.0 [6.0, 11.0]	0.006
Number of oocytes cleaved	9.0 [6.0, 11.0]	9.0 [6.0, 12.0]	8.00 [6.0, 11.0]	7.00 [5.0, 10.0]	0.003
Number of embryos available on Day3	4.0 [2.0, 7.0]	5.0 [3.0, 7.0]	4.0 [2.0, 6.0]	4.0 [2.0, 5.0]	0.012
Fertilization rate, %	87.5 [75.0, 100.0]	87.5 [73.3, 100.0]	83.3 [66.7, 93.8]	80.0 [60.0, 88.9]	< 0.001
Cleavage rate, %	100.0 [100.0, 100.0]	100.0 [100.0, 100.0]	100.0 [100.0, 100.0]	100.0 [100.0, 100.0]	0.490
D3 available embryos rate, %	50.0 [31.8, 70.0]	54.5 [33.3, 75.0]	50.00 [28.6, 70.0]	57.1 [31.7, 71.4]	0.592

*Abbreviation*: *BMI* body mass index. *Note*: Values are median (interquartile range) or number (%)

### Sperm DFI values and IVF outcomes

**Fig. 3:**
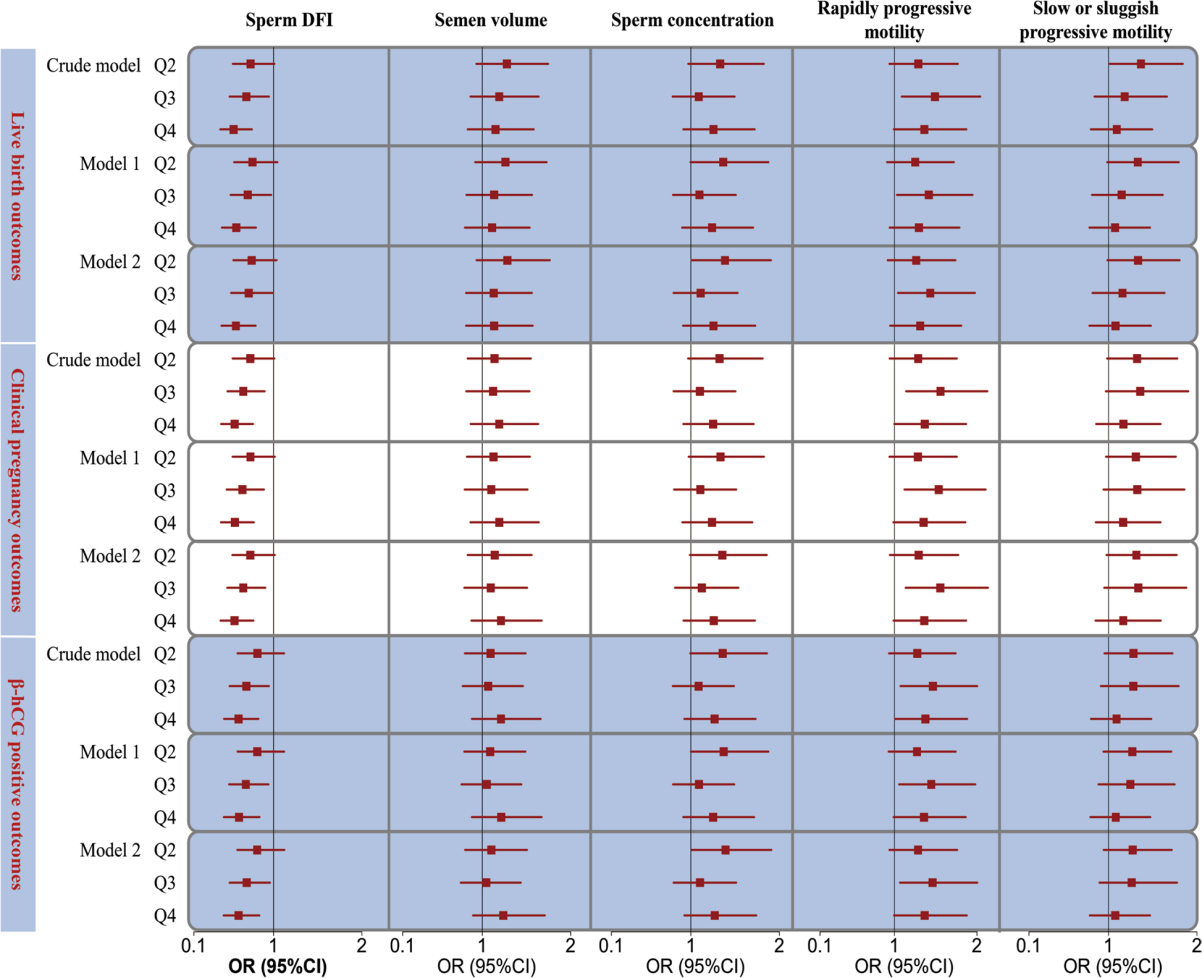
The forest plots of IVF outcomes in relation to the levels of sperm DFI and the studied routine semen parameters. Abbreviations: DFI, DNA fragmentation index; OR, odds ratio; CI, confidence interval; BMI, body mass index; AMH, Anti-Mullerian hormone; E2, Estradiol; FSH, Follicle-stimulating hormone. Notes: Model 1 was adjusted for duration of attempt to conceive, female age, male age, female BMI, and male BMI. Model 2 was further adjusted for controlled ovulation stimulation protocols, AMH, E2, FSH, endometrial thickness, and numbers of oocytes retrieved

After controlling for covariates such as the duration of the attempt to conceive, female age, male age, female BMI, male BMI, controlled ovulation stimulation protocols, AMH level, E_2_ level, FSH level, endometrial thickness, and the number of oocytes retrieved, linear exposure–response relationships were observed between the sperm DFI value and live birth, clinical pregnancy, and positive *β*-hCG outcomes (*P* for overall < 0.05, *P* for nonlinear > 0.05) (Supplementary Fig. 4 and Supplementary Table 3). The results showed a decreasing trend in the live birth, clinical pregnancy, and *β*-hCG positivity rates with increasing sperm DFI values. The results shown in Supplemental Fig. 5 suggest a U-shaped relationship between the DFI and miscarriage rate. According to ROC curve analysis (Supplementary Fig. 6), the area under the ROC curve for the sperm DFI and live birth, clinical pregnancy, and positive β-hCG outcomes were 0.56 (95% CI, 0.53–0.59), 0.56 (95% CI, 0.53–0.59), and 0.55 (95% CI, 0.52–0.58), respectively, with cut-off values of 8.70%, 11.14%, and 11.14%. Individuals in the third and fourth quartiles of DFI values were less likely to have better IVF outcomes (including live birth, clinical pregnancy, and positive β-hCG outcomes) than those in the lowest quartile after controlling for demographic characteristics and ovulation stimulation-related factors, although the significance was attenuated after adjusting for additional covariates in Models 1 and 2 (Fig. 3 and Supplementary Table 4).

### Studied routine semen parameter levels and IVF outcomes

After controlling for covariates such as the duration of the attempt to conceive, female age, male age, female BMI, male BMI, controlled ovulation stimulation protocols, AMH level, E_2_ level, FSH level, endometrial thickness, and the number of oocytes retrieved, a linear exposure–response relationship was observed between the rapidly progressive motility level and clinical pregnancy and positive β-hCG outcomes (*P* = 0.025, *P* = 0.040, respectively), whereas no such relationship was observed with the live birth outcome (*P* = 0.106) (Supplementary Fig. 4 and Supplementary Table 3). Although not statistically significant, it is clear (Supplementary Fig. 4) that the increase in the sperm concentration was conducive to better IVF outcomes. The odds of a good IVF outcome increased when the slow or sluggish progressive motility level was low, but when the slow or sluggish progressive motility level was too high, it led to a poor IVF outcome (see Supplementary Fig. 4). For rapidly progressive motility levels, individuals in the third quartile of had better IVF outcomes than those in the lowest quartile, although the significance was diminished after adjusting for covariates in Model 1 and Model 2 (Fig. 3 and Supplementary Table 4). For semen concentration, individuals in the second quartile had better live birth outcomes than those in the lowest quartile (OR = 1.38; 95% CI, 1.01–1.91) (Fig. 3 and Supplementary Table 4).

### Multivariable clusters and IVF outcomes

**Fig. 4:**
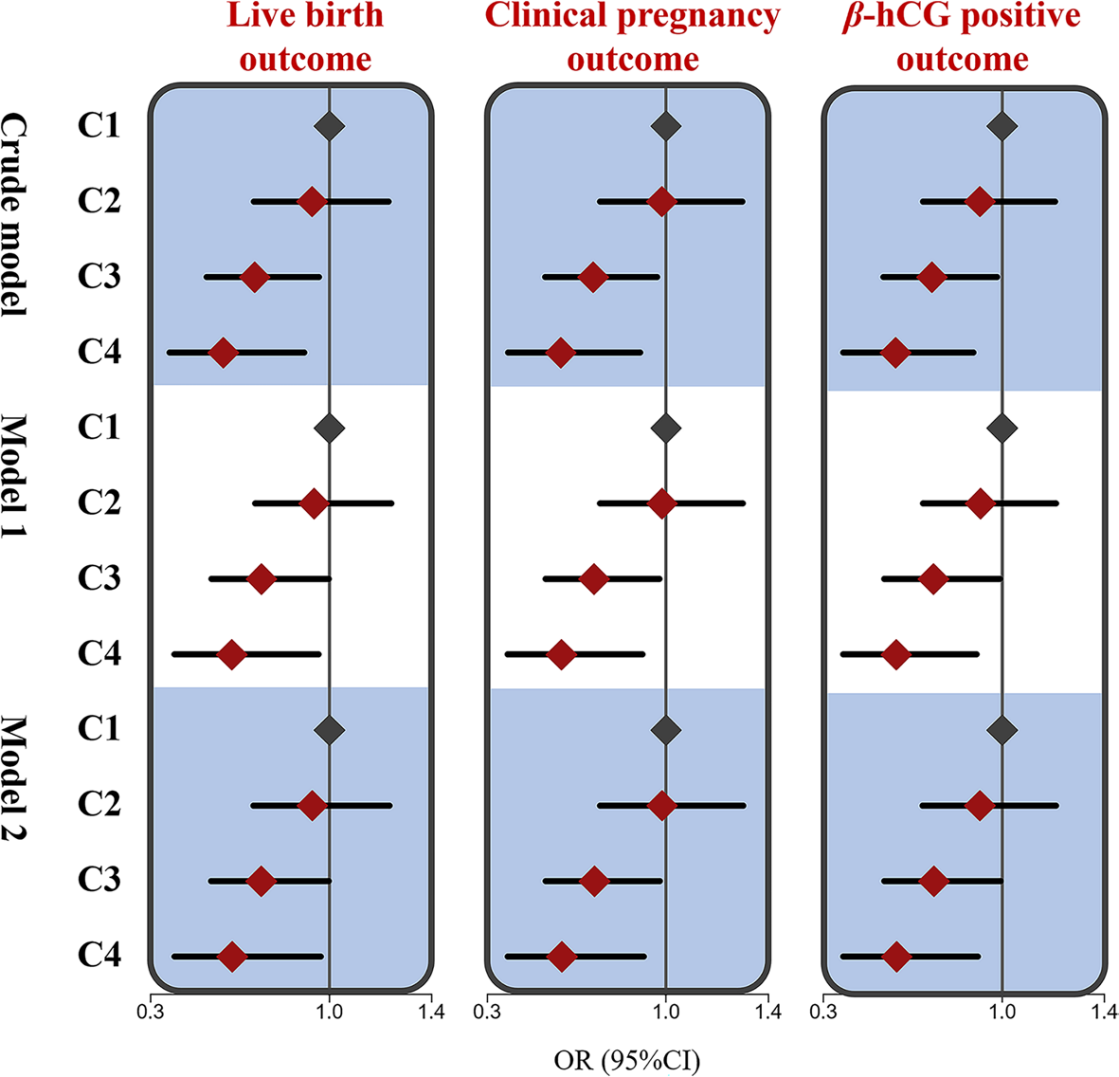
Results of live birth, clinical pregnancy, and β-hCG positive odds ratios (95%CI) across the 4 clusters. Model 1 was adjusted for duration of the attempt to conceive, female age, male age, female BMI, and male BMI; model 2 was additionally adjusted for controlled ovulation stimulation protocols, AMH, E2, FSH, endometrial thickness, and numbers of oocytes retrieved; C1, cluster 1 (low-level DFI/high-level semen parameter group); C2, cluster 2 (low-level DFI/median-level semen parameter group); C3, cluster 3 (low-level DFI/low-level semen parameter); C4, cluster 4 (high-level DFI/low-level semen parameter)

As the primary outcome, the live birth rates for the first fresh transfer IVF cycle were 47.7%, 45.9%, 39.2%, and 34.8% from Cluster 1 to Cluster 4, respectively (Table 2). No statistically significant differences in IVF outcomes were observed between Cluster 1 (low-level DFI/high-level semen parameter group) and Cluster 2 (low-level DFI/median-level semen parameter group). In Model 2, the odds of live birth, clinical pregnancy, and positive β-hCG outcomes were lower in Cluster 3 (low-level DFI/low-level semen parameter group) than in Cluster 1, with ORs (95% CI) of 0.733 (0.537, 0.998), 0.720 (0.530, 0.977), and 0.733 (0.539, 0.995), respectively. Compared with Cluster 1, Cluster 4 (high-level DFI/low-level semen parameter group) had even lower odds of live birth, clinical pregnancy, and positive β-hCG outcomes, with ORs (95% CI) of 0.620 (0.394, 0.967), 0.592 (0.381, 0.914), and 0.587 (0.379, 0.906), respectively, in Model 2. The results are provided in Table 2 and Fig. 4.

**Table 2:** The crude and multi-variate adjusted odds ratios (95% CIs) of IVF outcomes in relation to the multi-variable co-exposure clusters

**Cluster**	**Events/patients**	**Crude model**	**Model 1**^**a**^	**Model 2**^**b**^
***Live birth outcome***				
Cluster 1^c^	165/346	ref	ref	ref
Cluster 2^d^	215/468	0.932 (0.706, 1.232)	0.940 (0.710, 1.244)	0.933 (0.704, 1.236)
Cluster 3^e^	129/329	**0.708 (0.521, 0.960)**	**0.734 (0.539, 0.998)**	**0.733 (0.537, 0.998)**
Cluster 4^f^	40/115	**0.585 (0.375, 0.902)**	**0.618 (0.394, 0.959)**	**0.620 (0.394, 0.967)**
***Clinical pregnancy outcome***				
Cluster 1^c^	190/346	ref	ref	ref
Cluster 2^d^	255/468	0.983 (0.743, 1.299)	0.984 (0.743, 1.302)	0.984 (0.742, 1.303)
Cluster 3^e^	153/329	**0.714 (0.527, 0.966)**	**0.718 (0.529, 0.974)**	**0.720 (0.530, 0.977)**
Cluster 4^f^	48/115	**0.588 (0.382, 0.899)**	**0.590 (0.381, 0.908)**	**0.592 (0.381, 0.914)**
***β-hCG positive outcome***				
Cluster 1^c^	197/346	ref	ref	ref
Cluster 2^d^	256/468	0.913 (0.690, 1.208)	0.915 (0.690, 1.212)	0.913 (0.689, 1.211)
Cluster 3^e^	161/329	**0.725 (0.535, 0.981)**	**0.731 (0.539, 0.992)**	**0.733 (0.539, 0.995)**
Cluster 4^f^	50/115	**0.582 (0.379, 0.889)**	**0.585 (0.379, 0.900)**	**0.587 (0.379, 0.906)**

*Note*:

^a^Model 1 was adjusted for duration of attempt to conceive, female age, male age, female BMI, and male BMI

^b^Model 2 was additionally adjusted for controlled ovulation stimulation protocols, AMH, E2, FSH, endometrial thickness, and numbers of oocytes retrieved

^c^Low-level DFI/high-level semen parameter group

^d^Low-level DFI/median-level semen parameter group

^e^Low-level DFI/low-level semen parameter group

^f^High-level DFI/low-level semen parameter group

Bold demonstrates statistical significance

### Mediation analysis of the association of IVF outcomes with clusters and fertilization rates

Supplementary Table 5 presents the results of the mediation analyses of the association of IVF outcomes with clusters and fertilization rates, adjusted for demographic characteristics and ovulation stimulation-related factors. The estimated ACMEs in Cluster 3 and Cluster 4 were statistically significantly different from zero compared to those in Cluster 1 (for instance, -0.02 (-0.04 ~ 0.00) and -0.06 (-0.12 ~ -0.01) for the live birth outcome, respectively), although the estimated average direct and total effects were not. As an example of the live birth outcome, the proportion of the mediation effect was 24.8% (21.2% ~ 27.9%) and 44.1% (41.0% ~ 48.1%) in Cluster 3 and Cluster 4, respectively, compared with Cluster 1.

## Discussion

To our knowledge, this is the first report to examine the joint effect of the sperm DFI and traditional semen parameters on single fresh conventional IVF outcomes using the unsupervised K-means clustering method. We found that as sperm DFI values increased, the incidence of favourable IVF outcomes decreased accordingly. We also observed that lower sperm concentration and motility levels had an adverse effect on IVF outcomes. By considering the sperm DFI and routine semen parameters together, the participants were clustered into four groups. Using Cluster 1 (low sperm DFI values and high sperm motility and semen concentration levels) as a reference, Cluster 2 (low sperm DFI values and moderate sperm motility and semen concentration levels) was not significantly associated with clinical pregnancy and live birth outcomes, Cluster 3 (low sperm DFI values and low sperm motility and semen concentration levels) had adverse outcomes, and Cluster 4 (high sperm DFI values and low sperm motility and semen concentration levels) had the worst outcomes.

It has been well documented that even sperm with damaged DNA can form pronuclei at fertilization and continue subsequent embryonic development with the benefit of assisted reproductive technology, with theoretically detrimental consequences for assisted reproductive technology (ART) outcomes [32, 33]. However, in real-world studies (RWS), the impact of sperm DNA damage on ART outcomes remains partially controversial. Most studies suggest that sperm DNA damage has a detrimental effect on the clinical pregnancy outcome after conventional IVF [15–19]. Several meta-analyses [16, 18, 19] have shown that high sperm DFI groups were associated with lower pregnancy rates after conventional IVF with a relative risk (RR) ranging between 0.69 and 0.81. Our study revealed a negative linear correlation between DFI values and clinical pregnancy outcomes (as shown in Supplementary Table 3 and Supplementary Fig. 4), which was consistent with the results of these publications. In one meta-analysis, males with high sperm DFI values who underwent IVF and ICSI had a significantly reduced live birth rate, with a total OR of 1.17 (95% CI = 1.07–1.28, *P* < 0.001) [17]. A recently updated systematic review and meta-analysis showed a negative trend between sperm DFI values and the live birth rate in the case of IVF, although it was not statistically significant (RR = 0.48; 0.22–1.02; I^2^ = 79) [15]. Our study showed a negative linear relationship between DFI values and live birth outcomes in single fresh IVF cycles (*P* for overall = 0.007, *P* for nonlinear = 0.553). However, some studies have not identified a correlation between DFI values and IVF outcomes [20–22]. Cissen et al. [22] reviewed 30 studies to evaluate the utility of SDF in predicting the likelihood of continuing a pregnancy after IVF or ICSI and ultimately concluded that the DFI had limited ability to predict the chance of pregnancy in the context of ART. According to Esbert et al., [21] sperm DNA fragmentation was not related to the outcomes of IVF cycles.

One potential explanation for the negative results mentioned above [20–22] is that these studies neglected the effect of routine semen parameters and focused mainly on the association between the sperm DFI and IVF outcomes. The sperm DFI and conventional semen parameters are relatively independent of each other, as our study and some previous studies [34–36] have shown a low correlation coefficient between the two (the correlation coefficients ranged from -0.2 to 0.4). Standard semen parameters could also roughly predict the fertility potential of male factors and their impact on the outcomes of conventional IVF [6–9]. We evaluated the association of semen parameters with IVF outcomes in this study and found that participants with high sperm concentrations and rapidly progressive motility levels and in higher categories (e.g., rapidly progressive motility > 40% or in the third quartile) had higher odds of having good IVF outcomes, as we expected. This prompted the need for a combined assessment including the DFI as well as routine semen parameters.

Thus, in the present study, we defined four cluster patterns with different sperm DFI values and routine semen parameter levels to explore their combined effect on IVF outcomes. Our results revealed that live birth outcomes for fresh transfer IVF cycles were similar between the two groups when the sperm DFI value was at a lower level and routine semen parameters were at higher (Cluster 1) or intermediate levels (Cluster 2). However, even when the sperm DFI value remained at lower levels in previous studies [20, 25, 26, 37–39], low routine semen parameter levels were associated with decreased live birth rates. Moreover, our results also indicated that at worse DFI values and routine semen parameters levels, the live birth outcome was the worst of all clusters. Results similar to those described above were found for the clinical pregnancy outcome and the positive *β*-hCG outcome. Jr et al. had concerns about similar limitations and therefore included a population of men with normal routine semen parameters to assess the DFI and ART outcomes and still found a negative association between the two [39]. In 2021, the WHO published the Laboratory Manual for the Examination and Processing of Human Semen (6th edition), which highlights a new semen test, the sperm DNA fragmentation index, in Chapter 3, Section 2 (after routine semen and sperm morphological analysis) [5]. Both the manual and the findings of this study underline the importance of evaluating routine semen parameters and sperm DFI values in patients with male infertility. More importantly, treatment targets need to focus on both routine semen parameters and sperm DFI values for male infertility patients requiring IVF-assisted reproduction, according to our findings.

The selection of a standard female population and the control for female factors helped to identify the effect of the sperm DFI on IVF outcomes in a relatively unbiased manner. Jin et al. found that sperm DNA fragmentation has a negative impact on IVF and ICSI outcomes among women with reduced ovarian reserve (ROR) [20]. However, this finding needs to be considered against the risk of confounding bias. This association may also be related to the characteristics of the female population with ROR, as ROR tends to be accompanied by a decline in oocyte quality and an increase in female age (mean age over 35 years). Hence, considering previous study designs, a series of inclusion criteria were developed for the female population in this study to establish a "standard female population" [24–26].

Our study revealed differences in the fertilization rates between clusters, so we analysed whether the fertilization rates mediated the effect of different clusters on IVF outcomes. The median fertilization rate was found to be approximately between 20–50% (Supplementary Table 5). This result was partially corroborated by the finding that some meta-analyses showed a negative association between the DFI and IVF outcomes, while no association was seen for ICSI outcomes [16, 18, 19, 40]. However, this was a post hoc analysis, and the significance of the results needs to be considered with caution.

In the last 20 years, the TUNEL test, the sperm chromatin dispersion test (SCD), the comet test, and acridine orange flow cytometry have been commonly employed in assisted reproduction and andrology labs [5]. Previous meta-analyses, whether ROC curves were constructed or diagnostic ORs were reported, showed a fair predictive power of the various current assays [22, 41]. The present study reported an area under the ROC curve of AO FCM for IVF outcomes of approximately 0.55, with moderate sensitivity but poor specificity, which was consistent with the findings of previous studies [16, 22]. Therefore, in the clinical interpretation of these results, not only the DFI but also the comprehensive profile of the couple undergoing ART, such as the routine semen parameters, need to be considered.

The findings of the current study should be interpreted in the proper context. The female population was limited to the standard female population. Additionally, women with influencing factors such as a history of recurrent abortion, multiple failed implantations, and less than three eggs retrieved were excluded, meaning that the conclusions cannot be extrapolated to individuals with these conditions. Last, only couples undergoing single fresh IVF cycles were included in this study, and couples with multiple cycles were not included for the following reasons: first, to reduce the effect of variability in the course of ART treatment in some couples with different cycles, and second, to reduce the effect of psychological factors.

This study has several limitations. First, the study population was a retrospective cohort, making it difficult to avoid selection bias, and future consecutive prospective cohort studies are needed to confirm these findings. Second, the sperm DFI values and semen parameter levels were not measured after sperm processing on the day of egg retrieval, and we matched semen analysis and DFI data from the nearest IVF insemination date through the medical electronic data system, but this still resulted in measurement errors; in addition, in practice, DFI values and semen parameter levels are not clinically applicable when performed on the day of transplantation. Third, morphological parameters were not included in the study because a large proportion of missing values were found after exporting data, and we did not plan to perform manual entry to avoid human-generated errors.

## Conclusions

In conclusion, the combined effect of low sperm DFI values, high or moderate sperm concentrations, and sperm motility levels were associated with favourable IVF outcomes. Low sperm parameter levels, even when DFI values remain low, may still lead to poor IVF outcomes. Participants with high sperm DFI values and low routine semen parameter levels had the worst outcomes. Our findings offer a novel perspective for exploring the joint effects of the sperm DFI and routine semen parameter values.

## Supplementary Information

**Additional file 1: Supplementary Fig. 1**. Heat-map illustration of pairwise correlations of routine semen parameters and sperm DNA fragmentation index. **Supplementary Fig. 2**. The results of consensus clustering. **Supplementary Fig. 3.** Visualization of K-means clustering of 1258 infertile couples based on studied variables. **Supplementary Fig. 4.** Multivariable adjusted odds ratios for IVF outcomes according to levels of the sperm DFI and the studied semen routine parameters on a continuous scale. **Supplementary Fig. 5**. Multivariable adjusted odds ratios for miscarriage outcomes according to levels of the sperm DFI on a continuous scale. **Supplementary Fig. 6**. Receiver operating characteristic (ROC) curves for sperm DFI and IVF outcomes. **Supplementary Table 1**. Baseline characteristics of all participants in this study. **Supplementary Table 2**. Distributions of the routine semen parameters and sperm DNA fragmentation index after Min–Max scaling. **Supplementary Table 3.** *P*-values of overall and non-linear dose–response relationships of the sperm DFI and the studied routine semen parameters with IVF outcomes in adjustment of demographic characteristics and ovulation stimulation-related factors. **Supplementary Table 4**. The crude and multi-variate adjusted odds ratios (95% CIs) of the IVF outcomes in relation to levels of the sperm DFI and the studied semen routine parameters. **Supplementary Table 5**. Mediation analysis with IVF outcomes in association with clusters and fertilization rate.

## Data Availability

Some or all data sets generated during and/or analyzed during the present study are not publicly available but will be made available from the corresponding author upon reasonable request.

## Abbreviations

ART: Assisted reproductive technology
AMH: Anti-Müllerian hormone
AO FCM: Acridine orange flow cytometry
BMI: Body mass index
CIs: Confidence intervals
CC: Clomiphene citrate
CDF: Cumulative distribution function
CMA: Causal mediation analysis
DFI: DNA fragmentation index
E2: Estradiol
FSH: Follicle-stimulating hormone
GnRH: Gonadotropin-releasing hormone
hCG: Human chorionic gonadotropin
IVF: In vitro fertilization
IQR: Interquartile range
ICSI: Intracytoplasmic sperm injection
LE: Letrozole
ORs: Odds ratios
ROC: Receiver operating characteristic
ROR: Reduced ovarian reserve
RR: Relative risk
RWS: Real-world studies
SCD: Sperm chromatin dispersion
