# Supplementary material for: Machine learning-based clustering to identify the combined effect of the DNA fragmentation index and conventional semen parameters on in vitro fertilization outcomes
Source: Reprod Biol Endocrinol. 2023 Mar 15;21:26. doi: 10.1186/s12958-023-01080-y (PMC10015711; doi:10.1186/s12958-023-01080-y)
Supplement: Supplementary file 1 — Additional file 1: Supplementary Fig. 1. Heat-map illustration of pairwise correlations of routine semen parameters and sperm DNA fragmentation index. Supplementary Fig. 2. The results of consensus clustering. Supplementary Fig. 3. Visualization of K-means clustering of 1258 infertile couples based on studied variables. Supplementary Fig. 4. Multivariable adjusted odds ratios for IVF outcomes according to levels of the sperm DFI and the studied semen routine parameters on a continuous scale. Supplementary Fig. 5. Multivariable adjusted odds ratios for miscarriage outcomes according to levels of the sperm DFI on a continuous scale. Supplementary Fig. 6. Receiver operating characteristic (ROC) curves for sperm DFI and IVF outcomes. Supplementary Table 1. Baseline characteristics of all participants in this study. Supplementary Table 2. Distributions of the routine semen parameters and sperm DNA fragmentation index after Min–Max scaling. Supplementary Table 3. P-values of overall and non-linear dose–response relationships of the sperm DFI and the studied routine semen parameters with IVF outcomes in adjustment of demographic characteristics and ovulation stimulation-related factors. Supplementary Table 4. The crude and multi-variate adjusted odds ratios (95% CIs) of the IVF outcomes in relation to levels of the sperm DFI and the studied semen routine parameters. Supplementary Table 5. Mediation analysis with IVF outcomes in association with clusters and fertilization rate. [file 12958_2023_1080_MOESM1_ESM.docx]

**Supplementary materials**

[**Supplementary Fig. 1** Heat-map illustration of pairwise correlations of routine semen parameters and sperm DNA fragmentation index. 2](#_Toc120696245)

[**Supplementary Fig. 2** The results of consensus clustering. 3](#_Toc120696246)

[**Supplementary Fig. 3** Visualization of K-means clustering of 1258 infertile couples based on studied variables. 5](#_Toc120696247)

[**Supplementary Fig. 4** Multivariable adjusted odds ratios for IVF outcomes according to levels of the sperm DFI and the studied semen routine parameters on a continuous scale. 6](#_Toc120696248)

[**Supplementary Fig. 5** Multivariable adjusted odds ratios for miscarriage outcomes according to levels of the sperm DFI on a continuous scale. 8](#_Toc120696249)

[**Supplementary Fig. 6** Receiver operating characteristic (ROC) curves for sperm DFI and IVF outcomes. 9](#_Toc120696250)

[**Supplementary Table 1** Baseline characteristics of all participants in this study. 10](#_Toc120696251)

[**Supplementary Table 2** Distributions of the routine semen parameters and sperm DNA fragmentation index after Min-Max scaling. 11](#_Toc120696252)

[**Supplementary Table 3** P-values of overall and non-linear dose-response relationships of the sperm DFI and the studied routine semen parameters with IVF outcomes in adjustment of demographic characteristics and ovulation stimulation-related factors. 12](#_Toc120696253)

[**Supplementary Table 4** The crude and multi-variate adjusted odds ratios (95% CIs) of the IVF outcomes in relation to levels of the sperm DFI and the studied semen routine parameters. 13](#_Toc120696254)

[**Supplementary Table 5** Mediation analysis with IVF outcomes in association with clusters and fertilization rate. 16](#_Toc120696255)

| 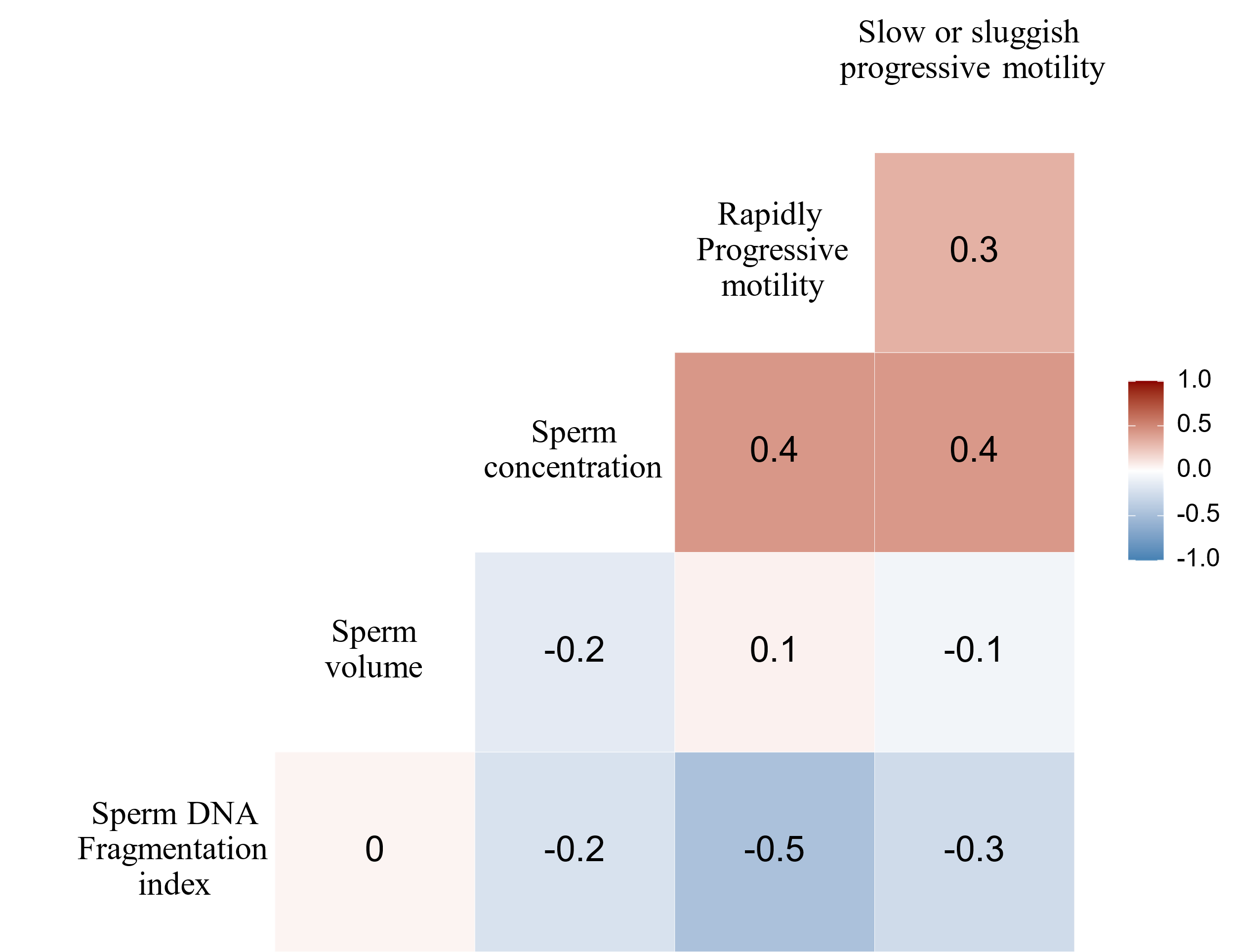 |
| --- |
| **Supplementary Fig. 1** Heat-map illustration of pairwise correlations of routine semen parameters and sperm DNA fragmentation index. |

| **A**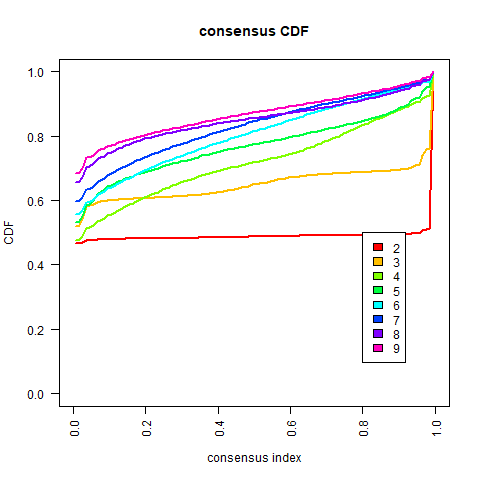 | **B**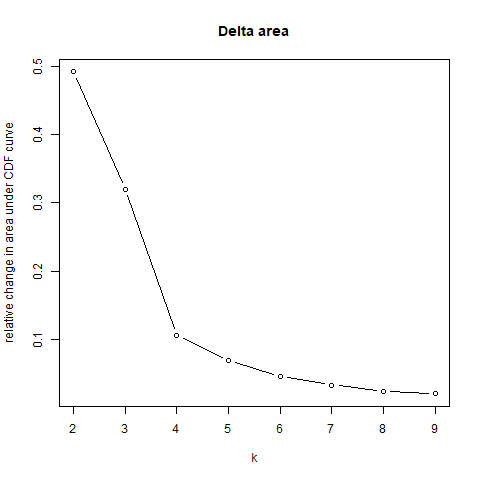 |
| --- | --- |
| **C**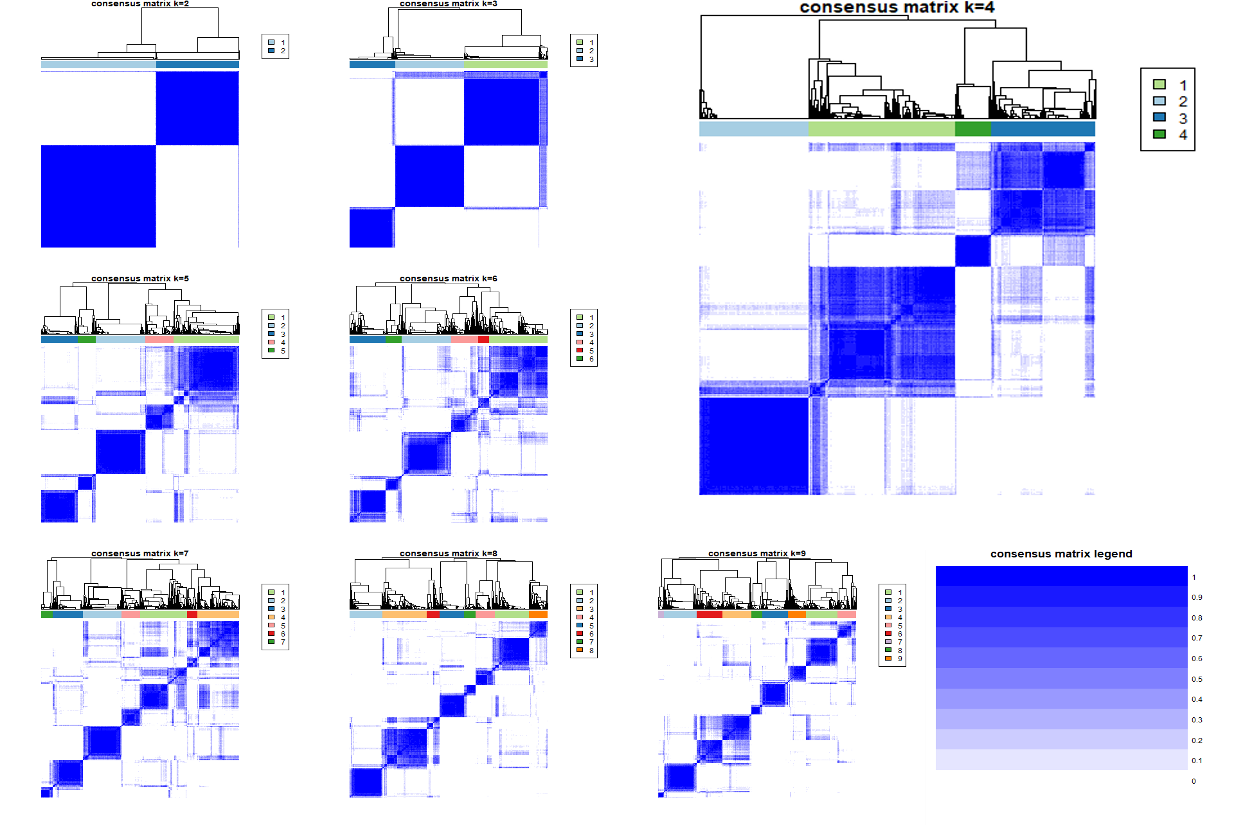 | |
| **D**  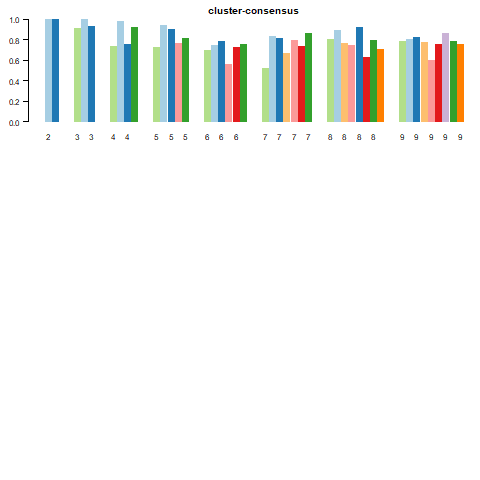 | |
| **Supplementary Fig. 2** The results of consensus clustering.  **Notes:** The cumulative distribution function (CDF) graph shows the consensus distribution of each cluster (A). The delta area plot displays the relative change in the area under the CDF curve (B). The maximum change in the area occurs between K = 3 and K = 5 when the relative increase in the area becomes significantly smaller. As shown in the CM heat map (C), cluster 2 and cluster 3 identified by the K-means algorithm have clear boundaries, and cluster 4 has relatively clear boundaries, indicating good cluster stability in repeated iterations. The mean cluster consensus score was comparable between a scenario of two, three, or four clusters (D). | |

| 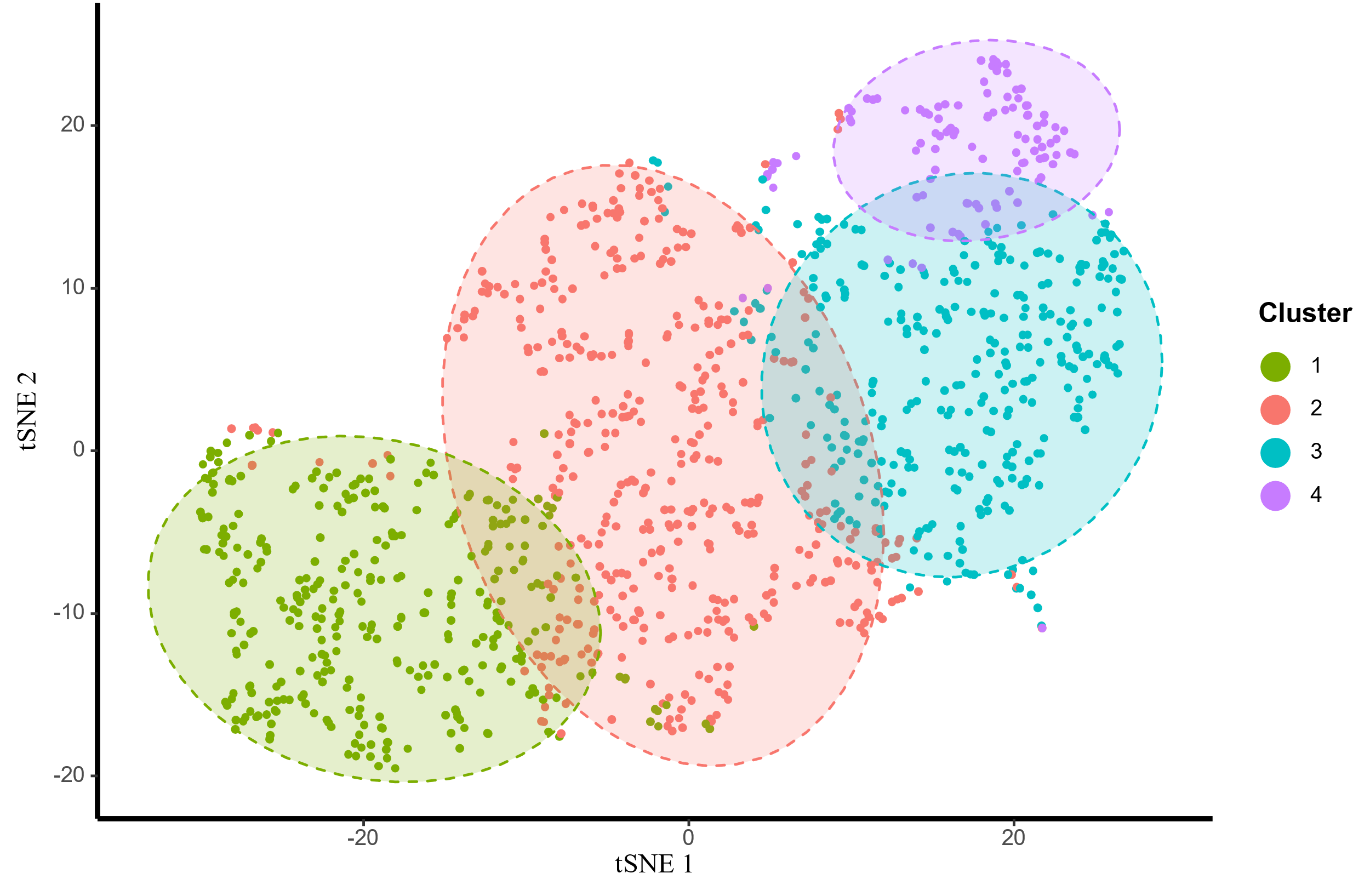 |
| --- |
| **Supplementary Fig. 3** Visualization of K-means clustering of 1258 infertile couples based on studied variables.  **Notes:** Green dots refer to cluster 1 (low-level DFI/high-level semen parameter group); red dots refer to cluster 2 (low-level DFI/median-level semen parameter group); blue dots refer to cluster 3 (low-level DFI/low-level semen parameter); purple dots refer to cluster 4 (high-level DFI/low-level semen parameter). |

| **Live birth outcomes** | **Clinical pregnancy outcomes** | **β-hCG positive outcomes** |
| --- | --- | --- |
| 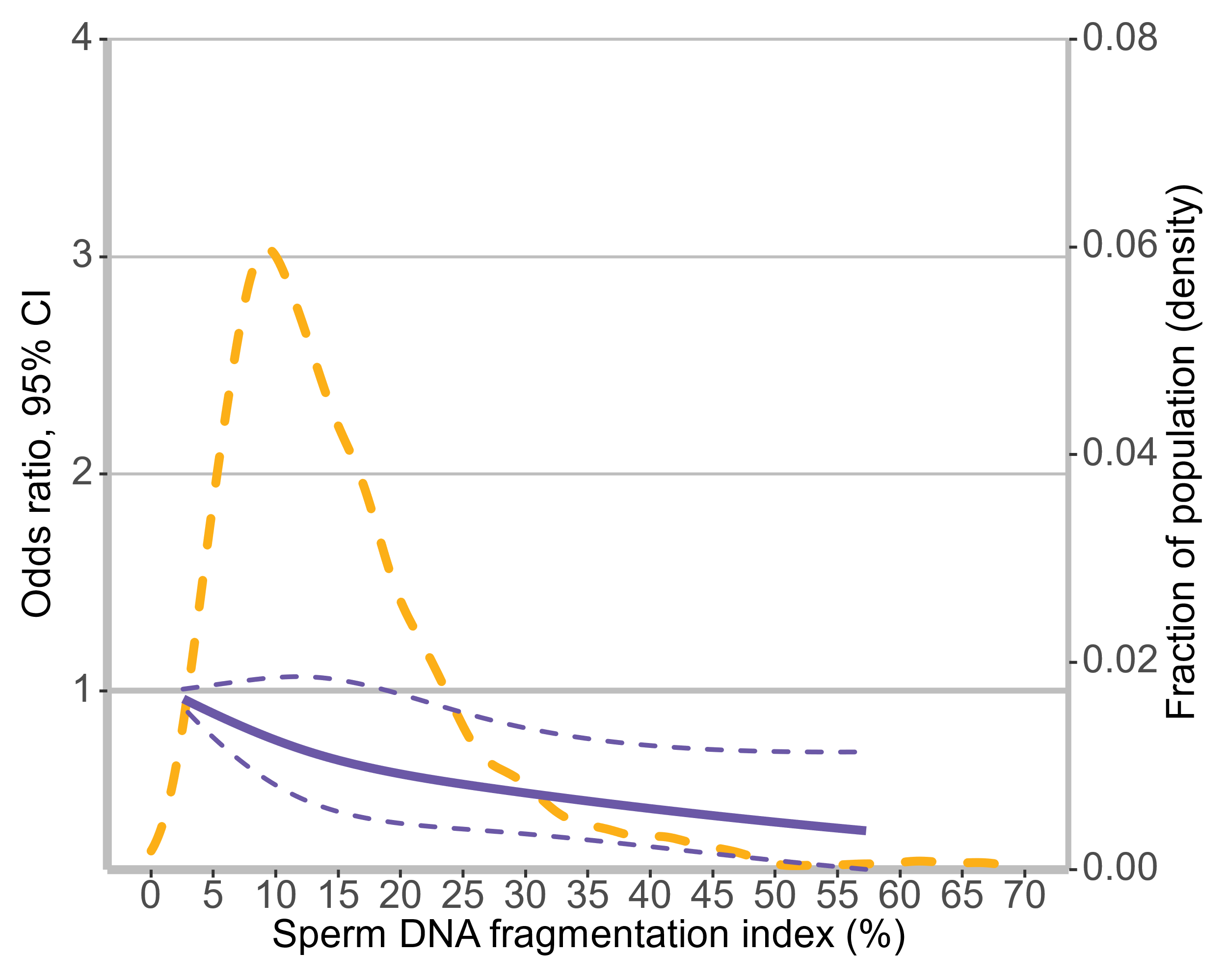 | 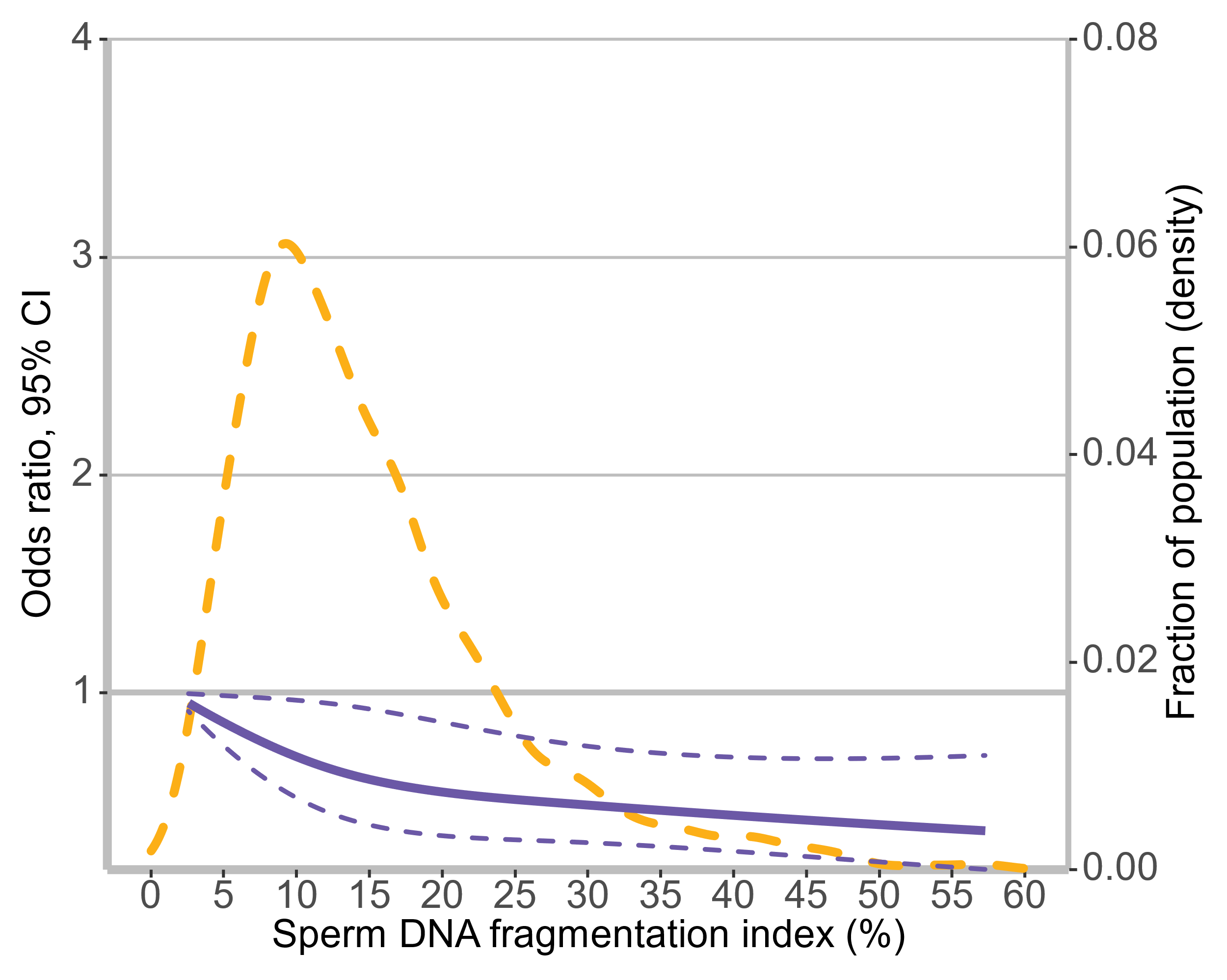 | 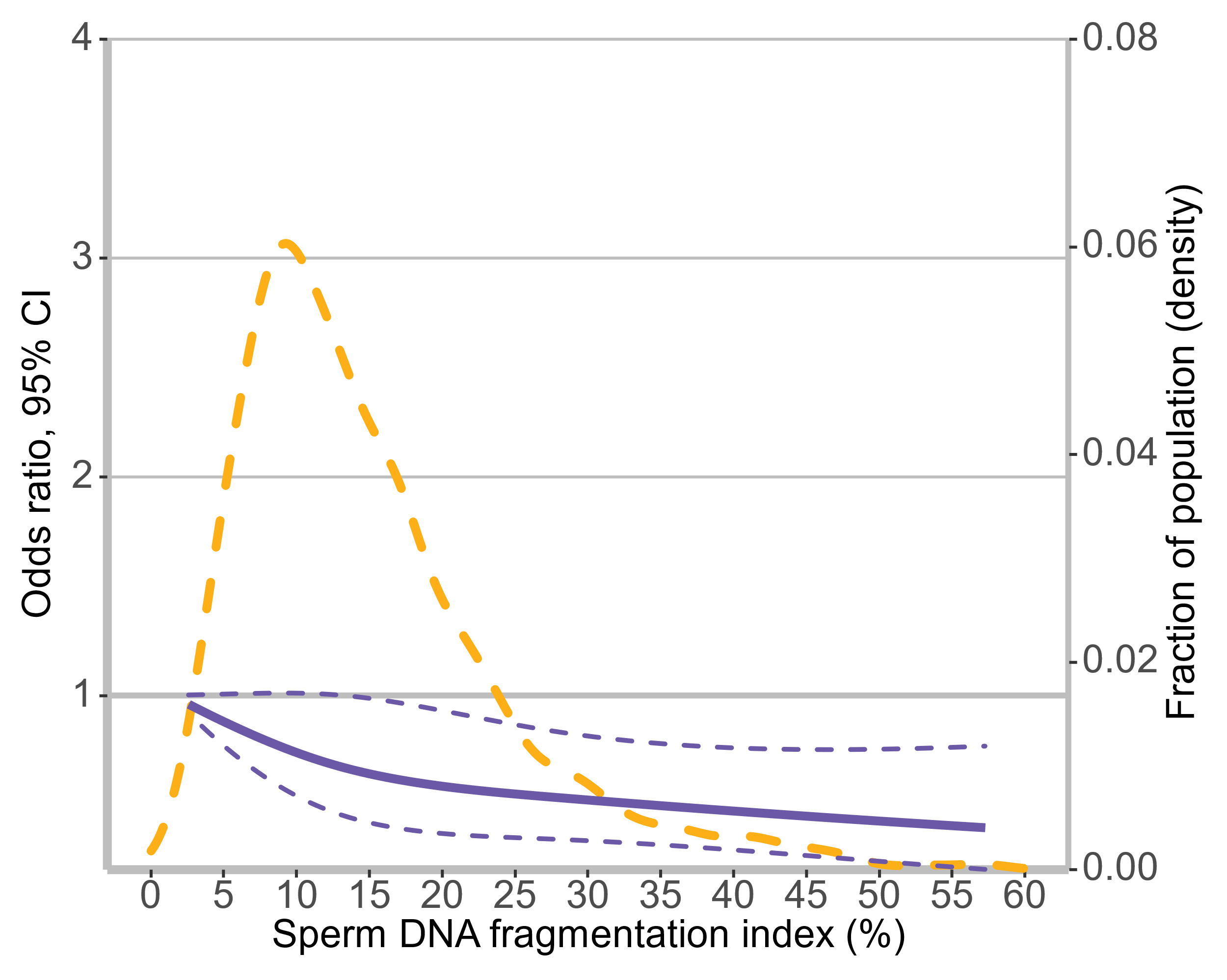 |
| 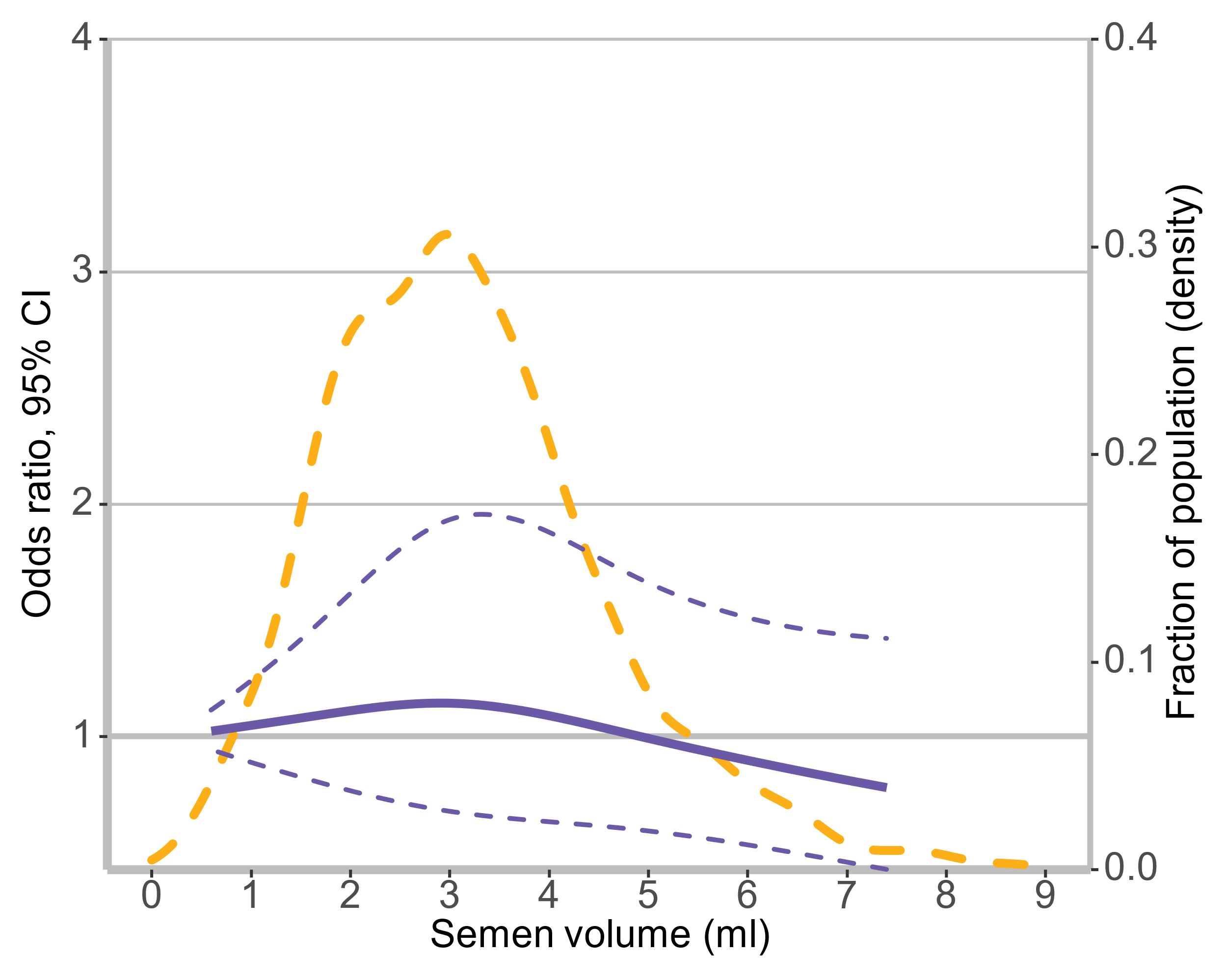 | 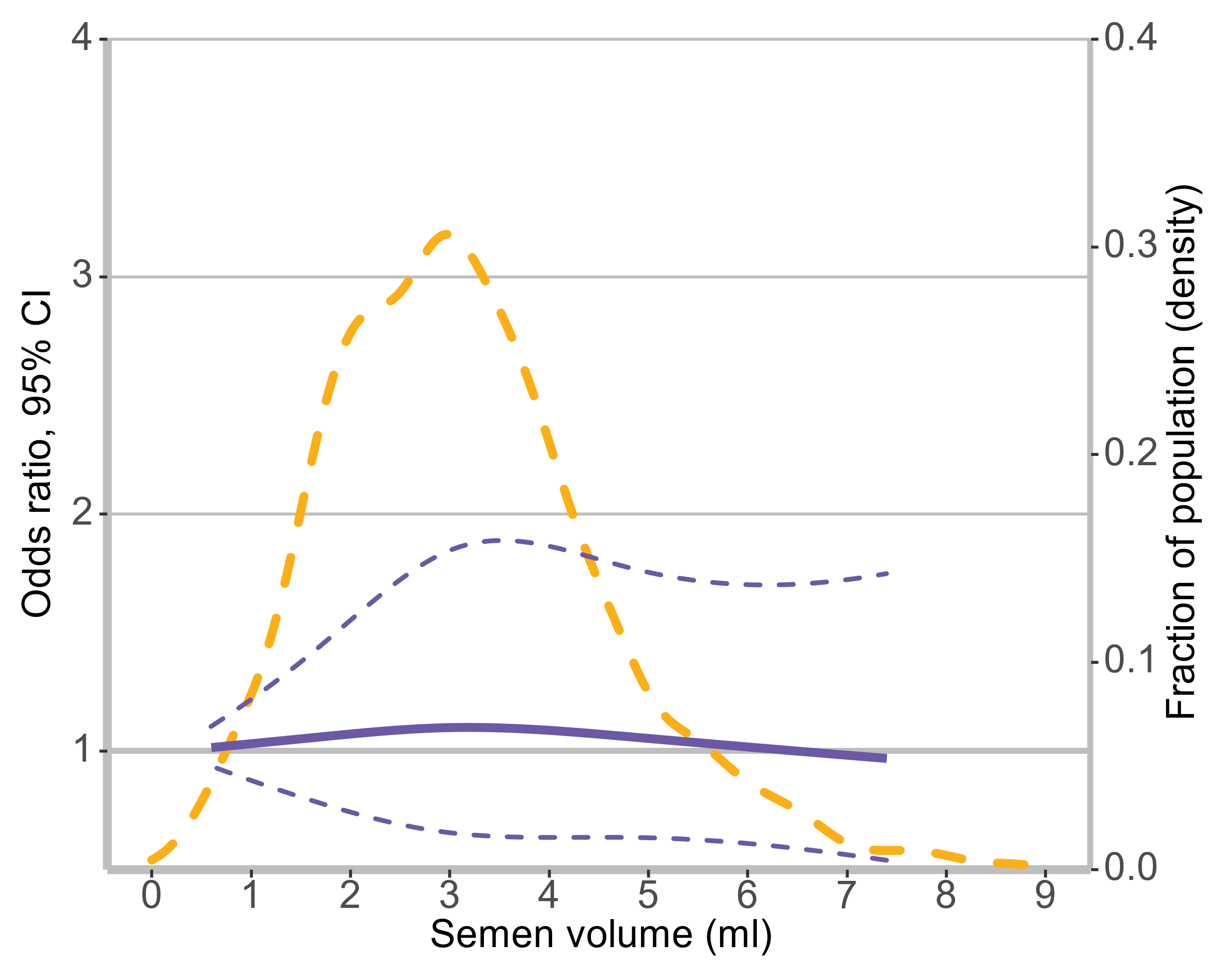 | 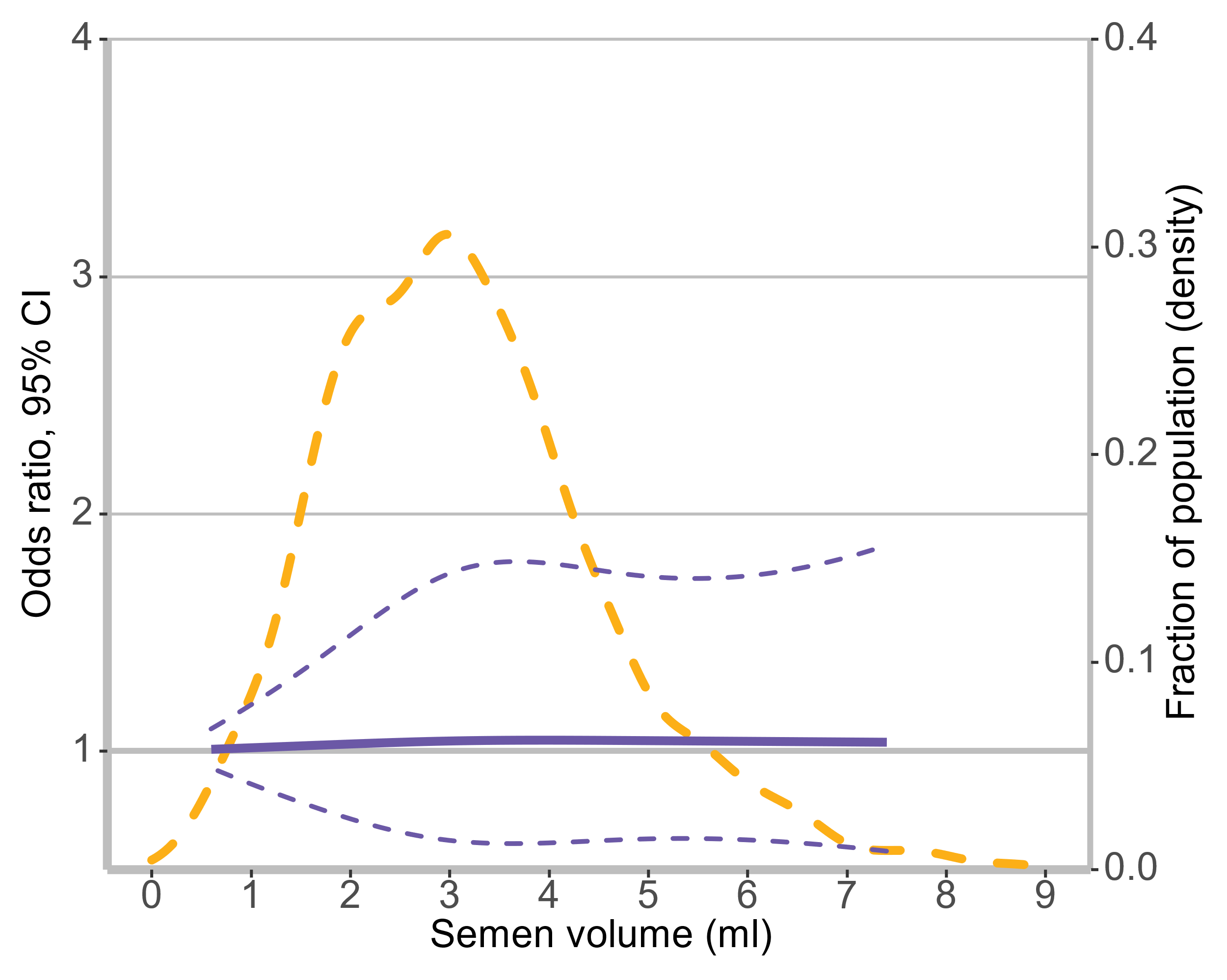 |
| 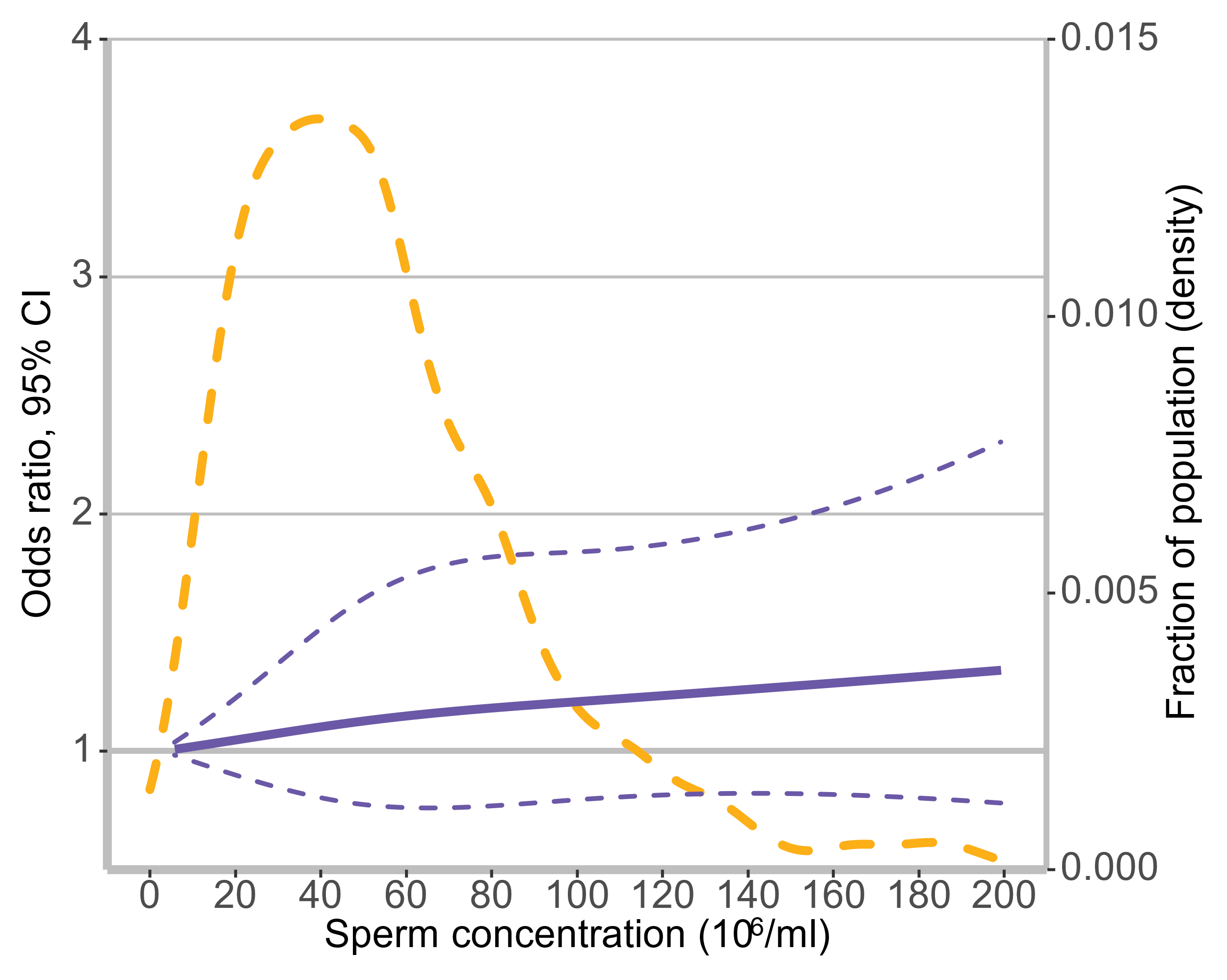 | 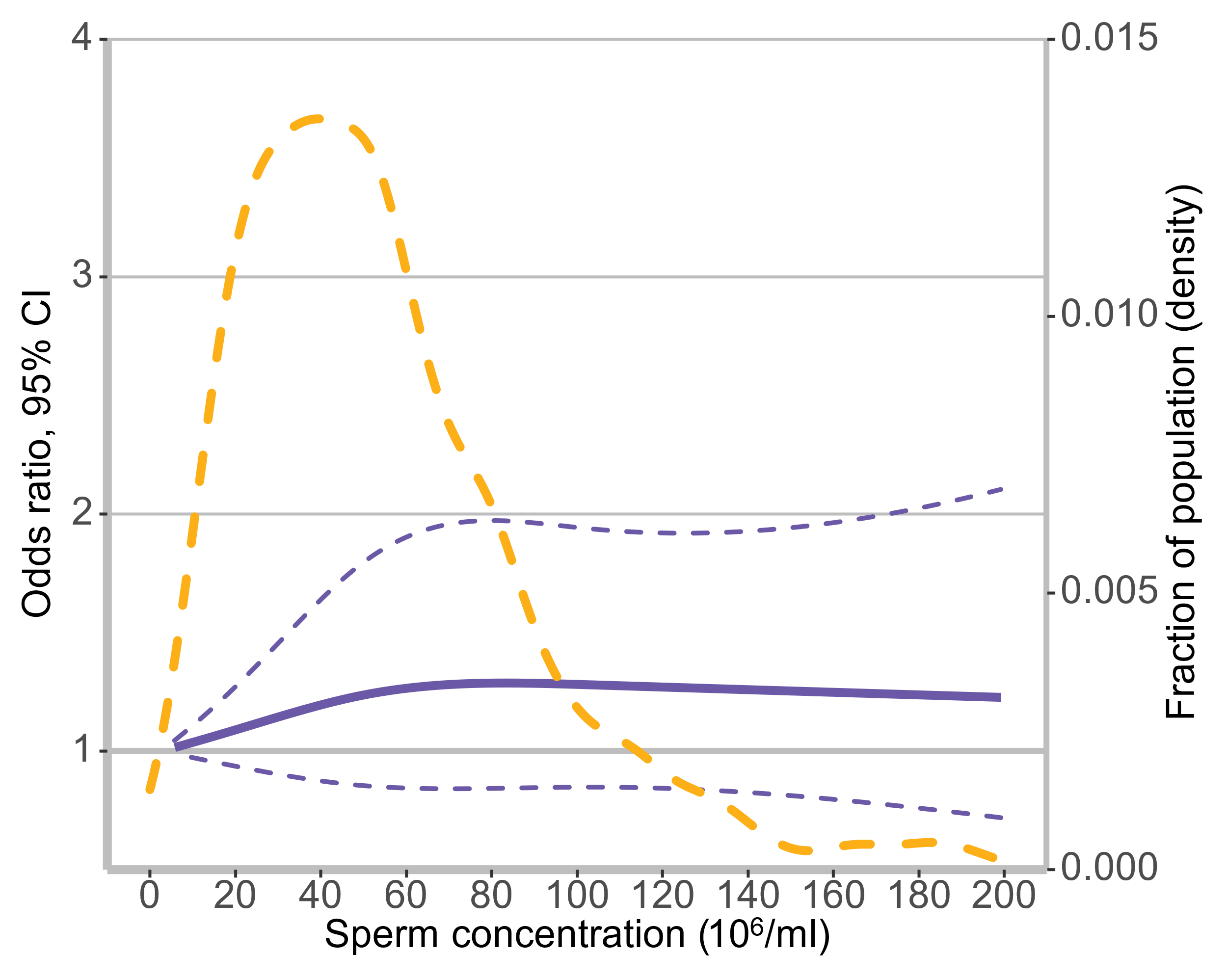 | 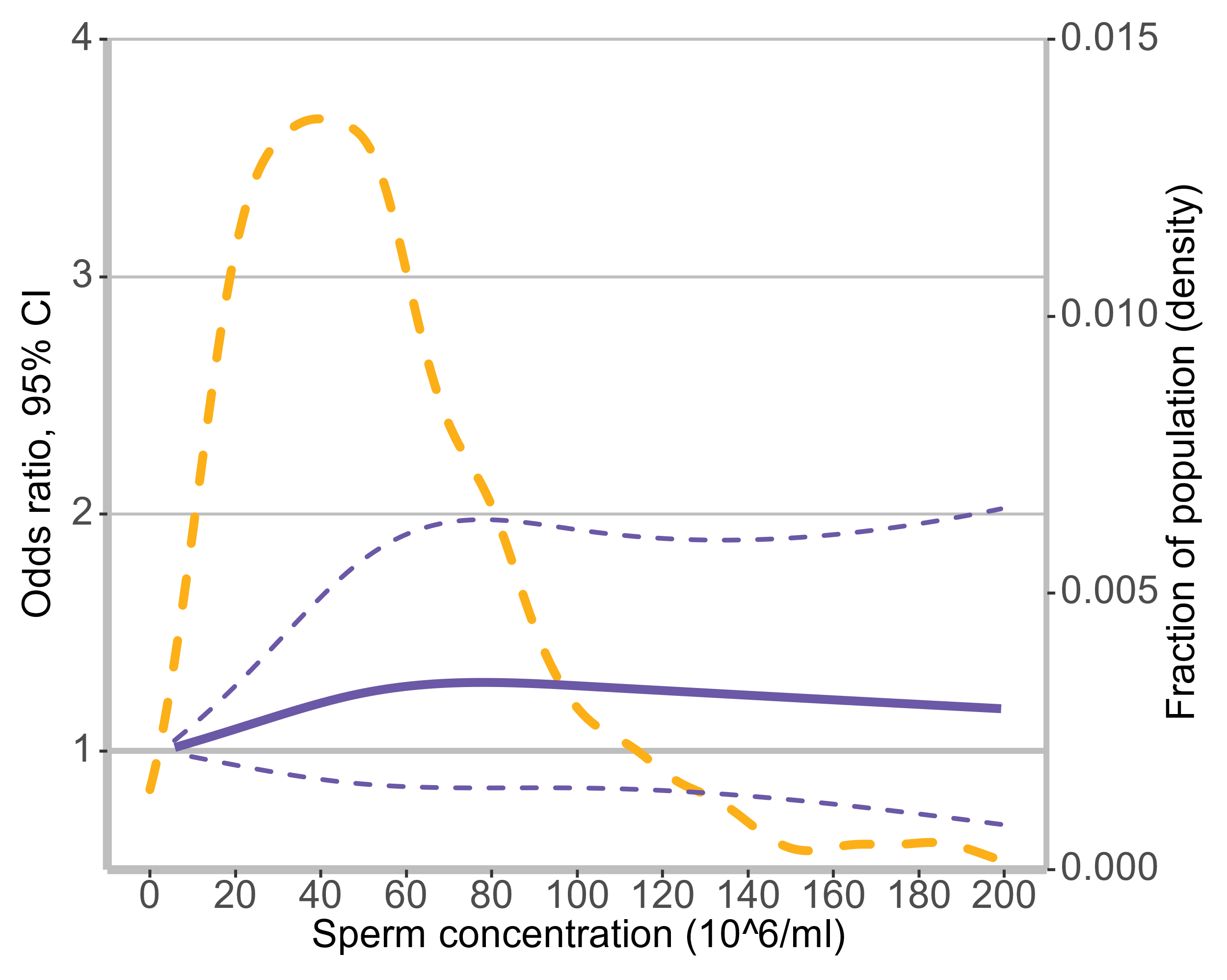 |
| 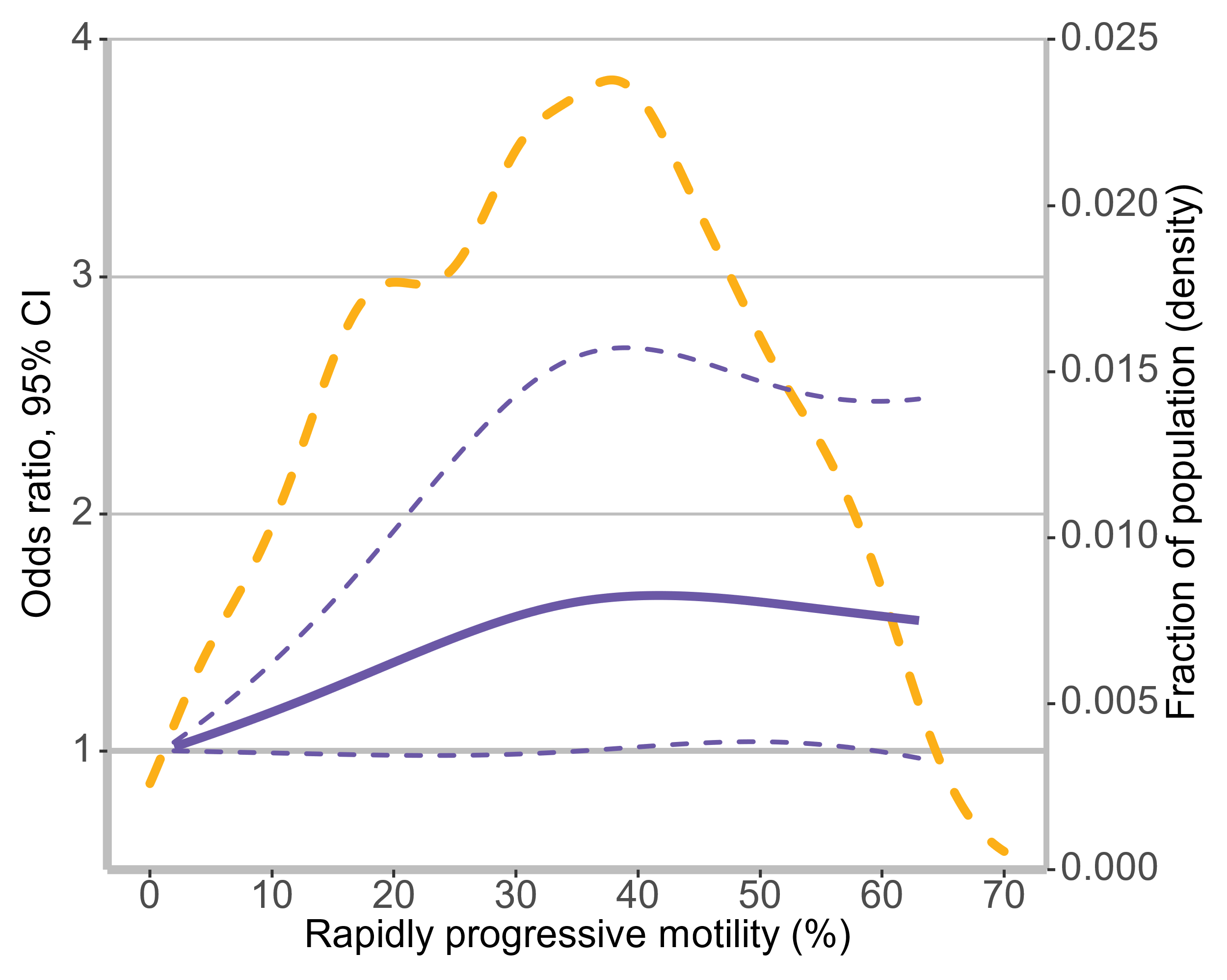 | 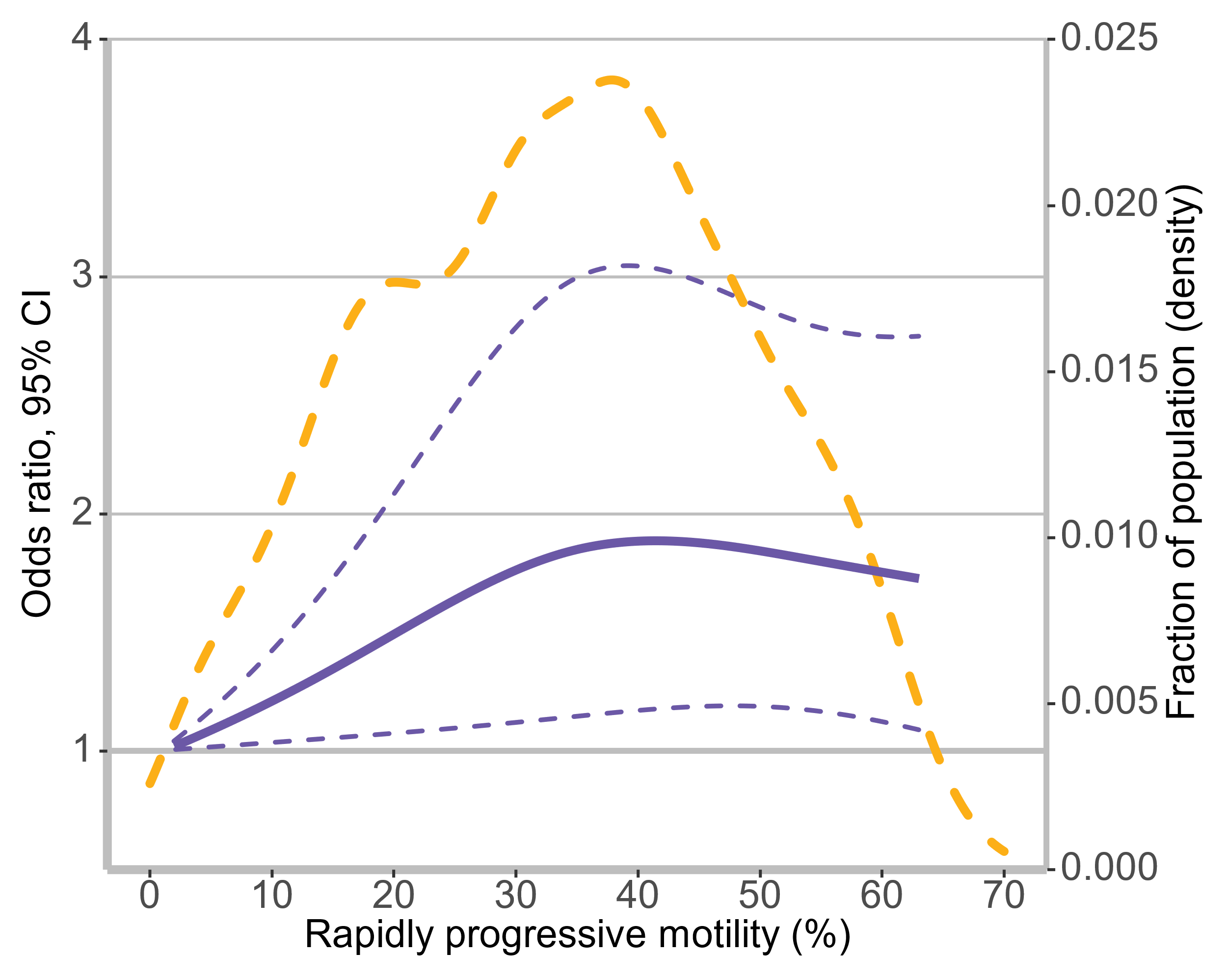 | 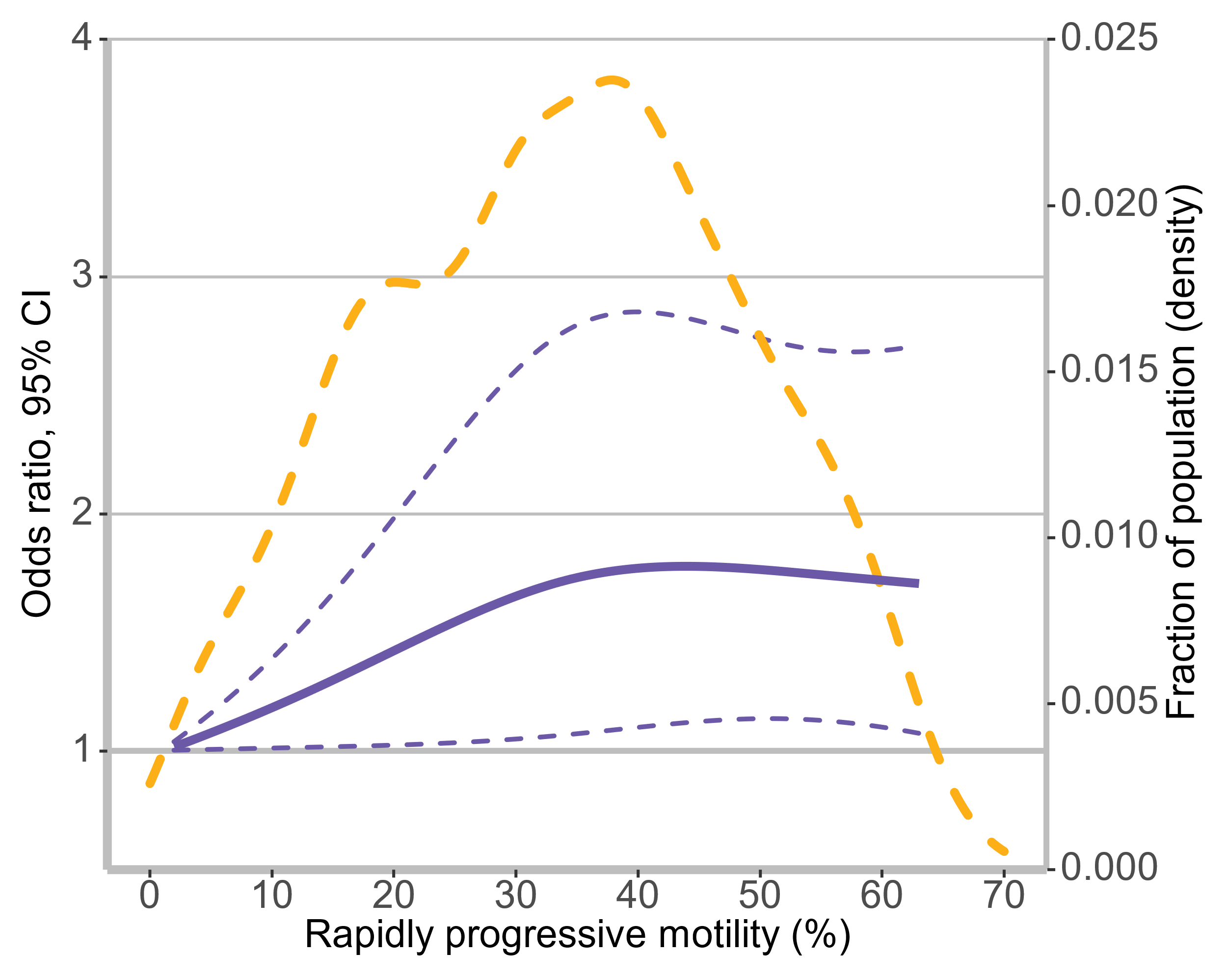 |
| 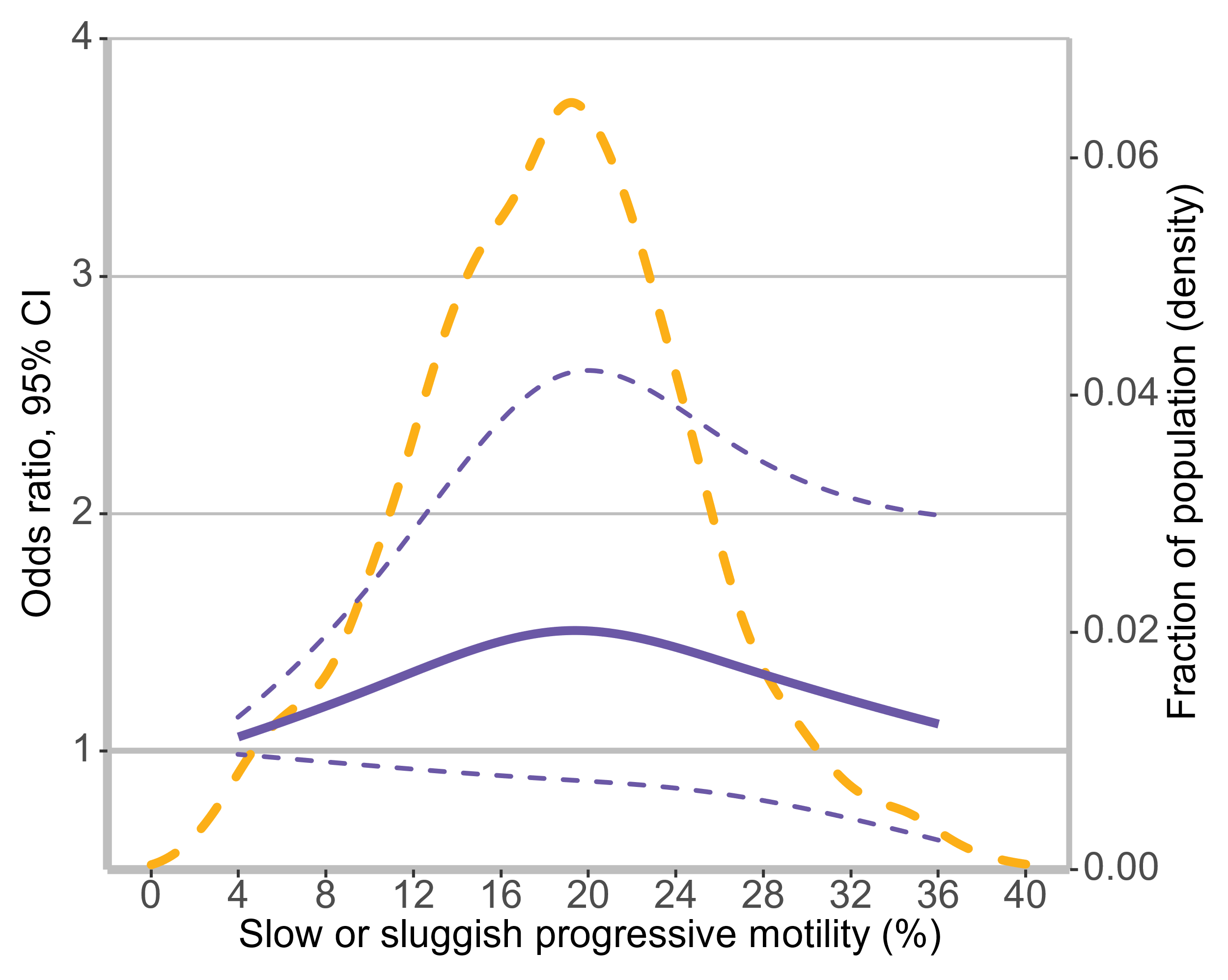 | 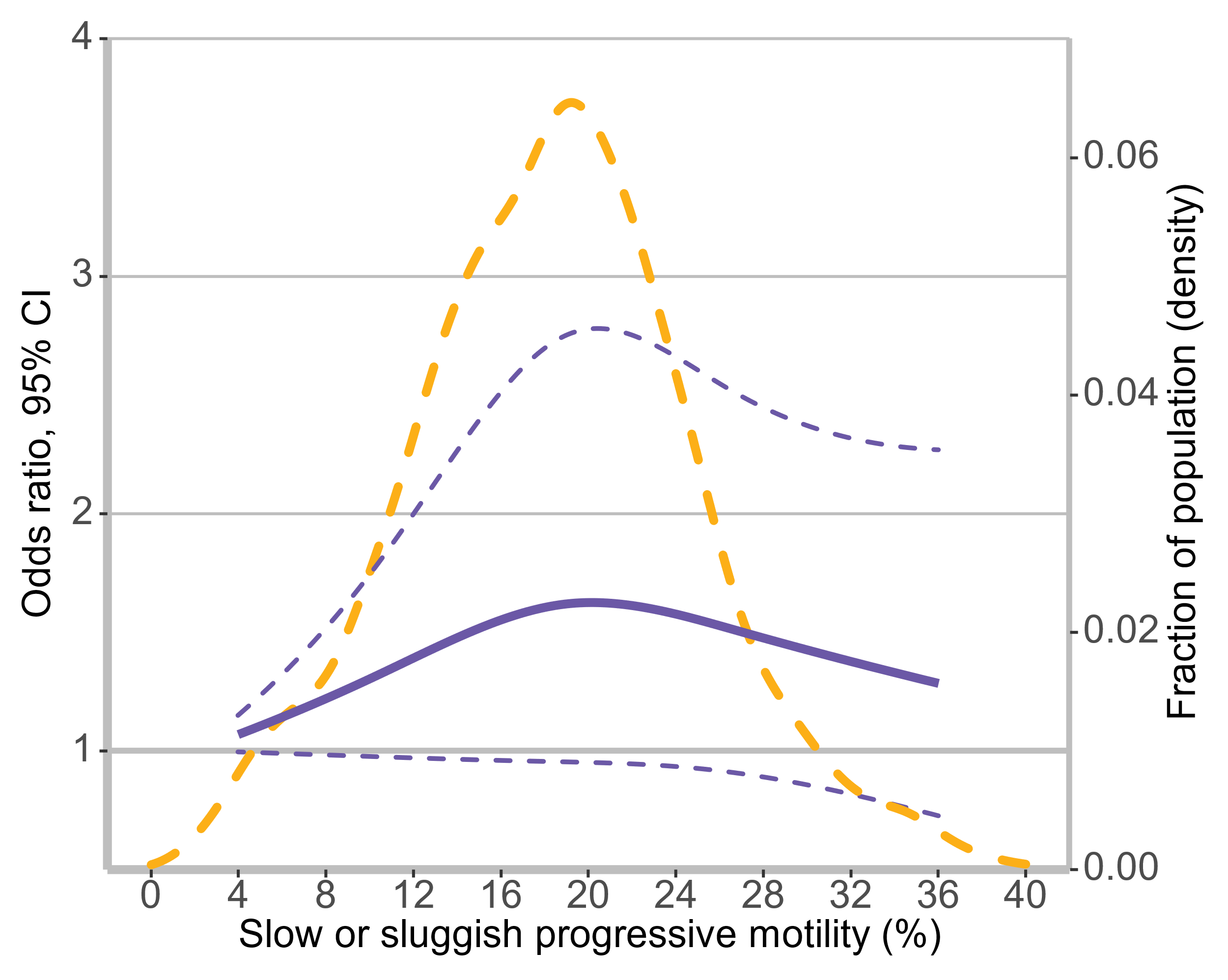 | 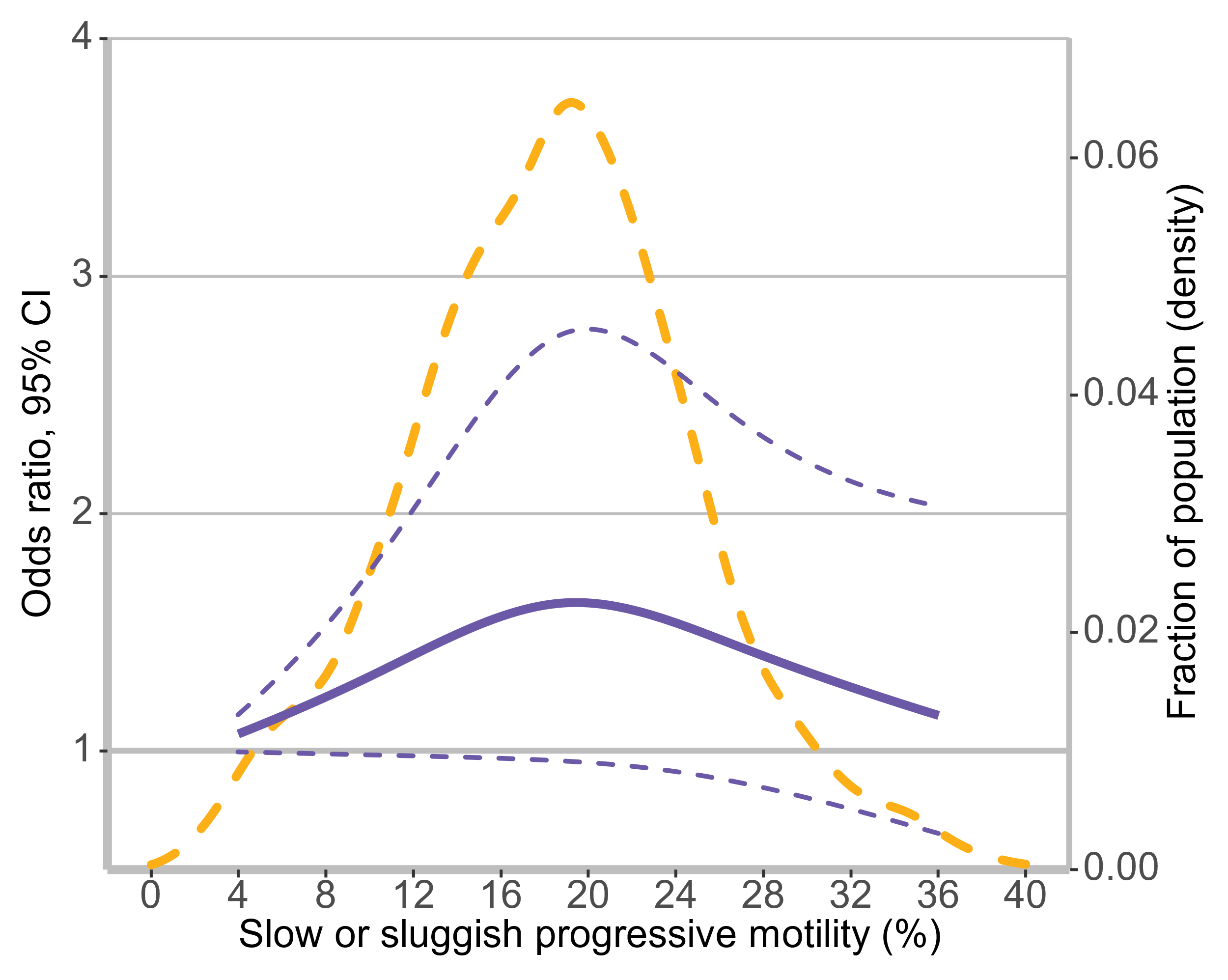 |
| **Supplementary Fig. 4** Multivariable adjusted odds ratios for IVF outcomes according to levels of the sperm DFI and the studied semen routine parameters on a continuous scale.  Notes: The blue solid line is the multivariate adjusted odds ratio, and the blue dashed line indicates the 95% confidence interval derived from the restricted cubic spline regression with three knots. The solid bold line indicates the case where the odds ratio is 1.0. Dashed yellow curves show the fraction of the population with different levels of the variable. Analyses were adjusted for duration of attempt to conceive, female age, male age, female BMI, male BMI, COS, AMH, E_2_, FSH, endometrial thickness, and numbers of oocytes retrieved. | | |

| 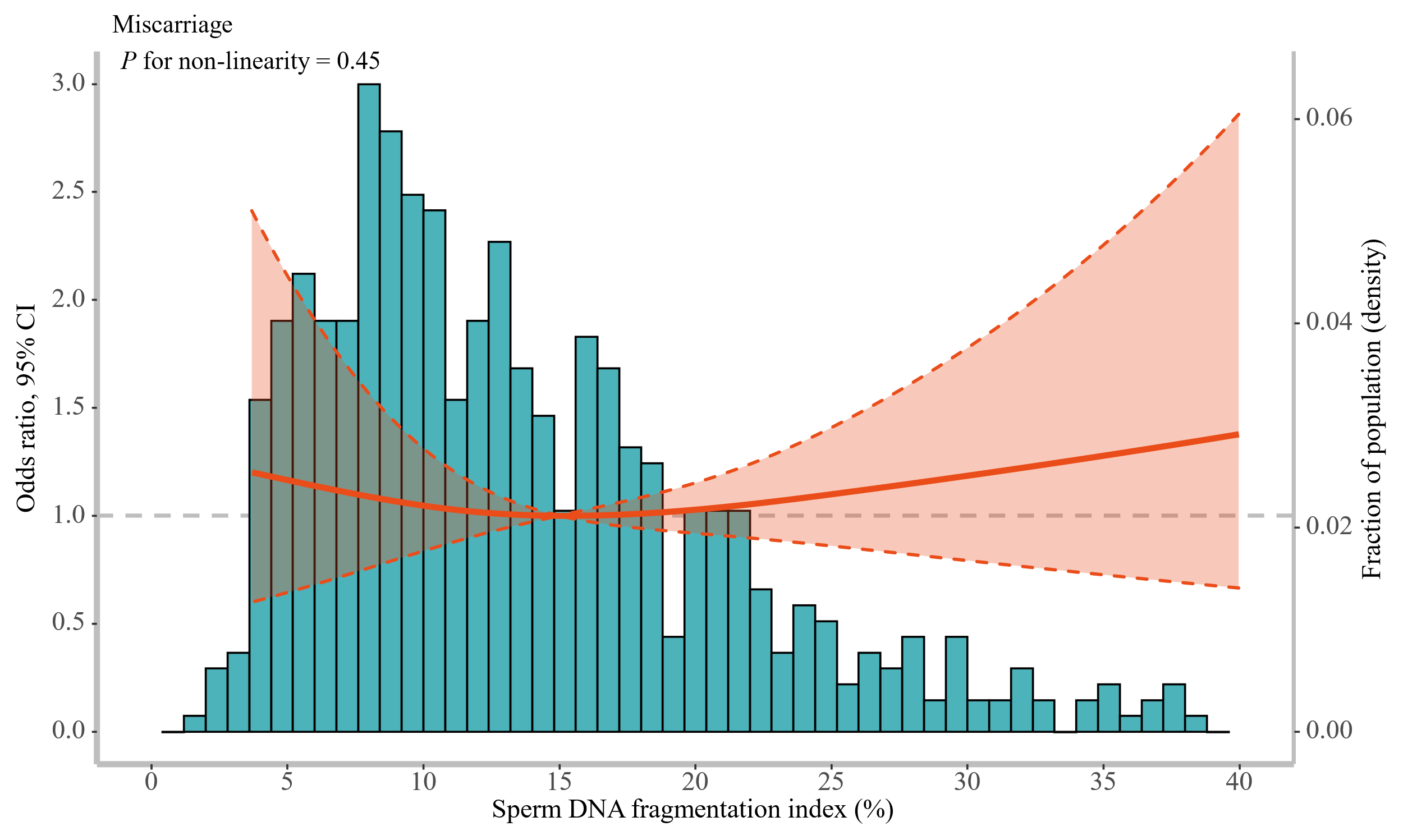 |
| --- |
| **Supplementary Fig. 5** Multivariable adjusted odds ratios for miscarriage outcomes according to levels of the sperm DFI on a continuous scale. |

| 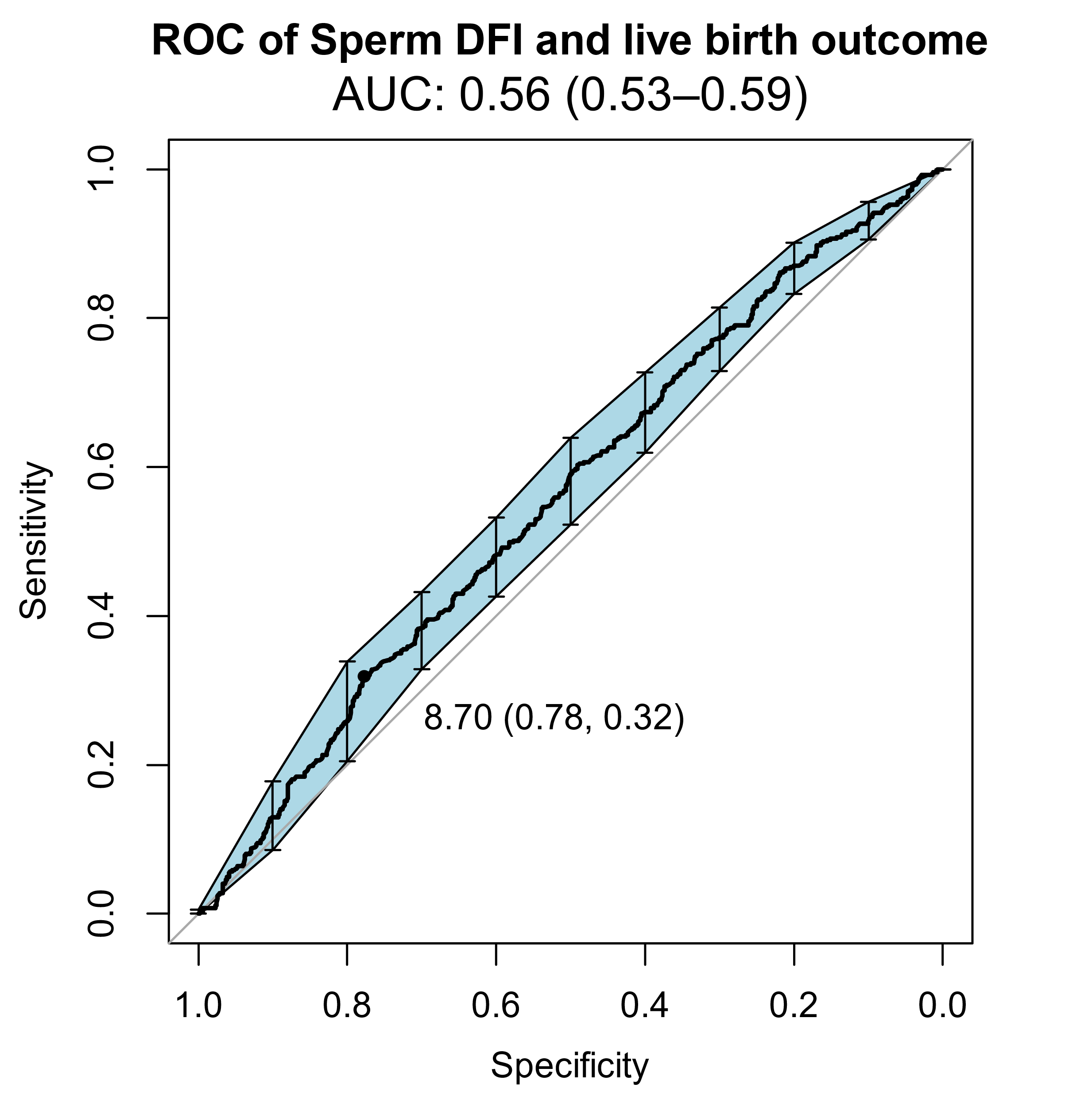 | 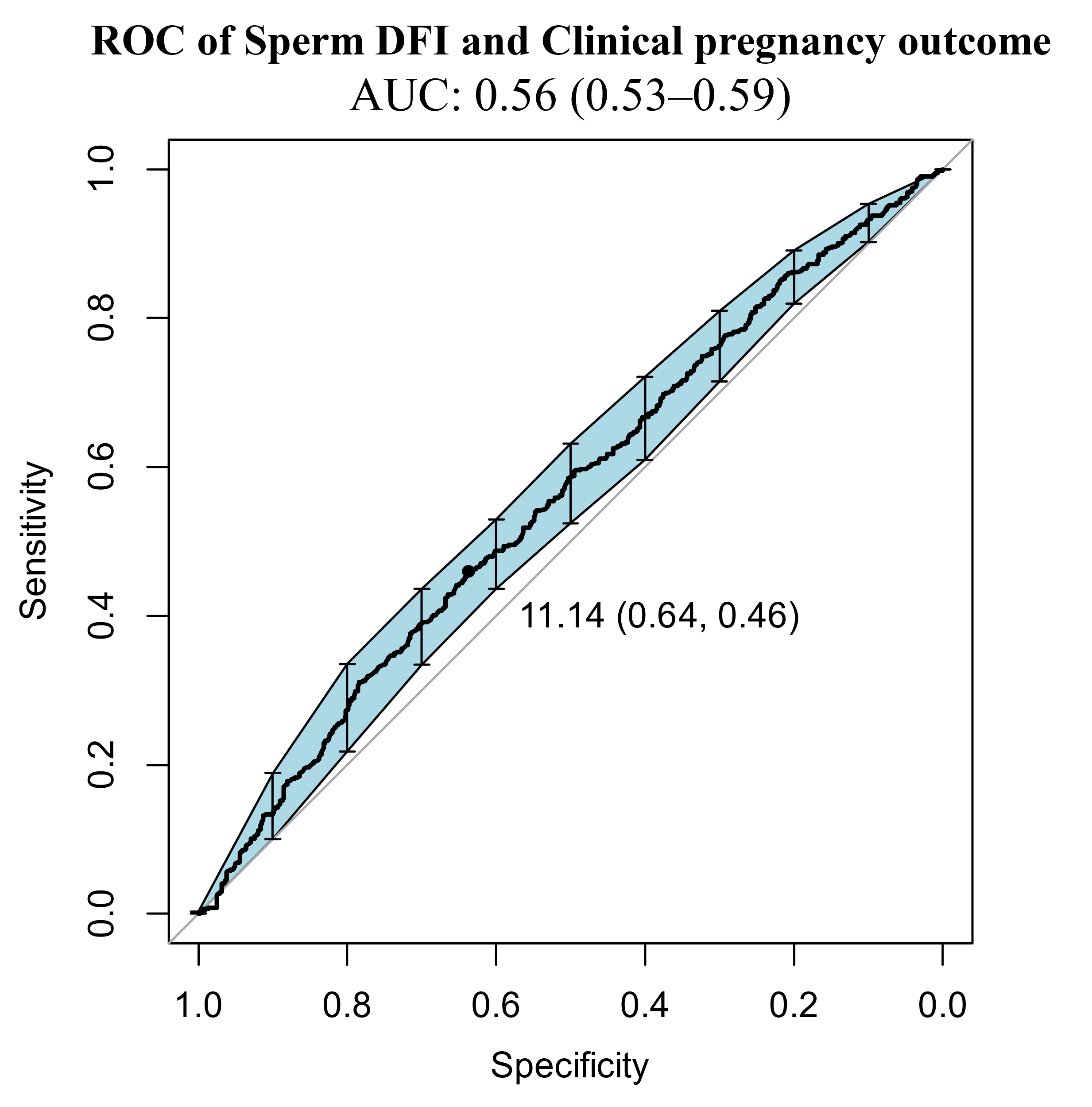 |
| --- | --- |
| 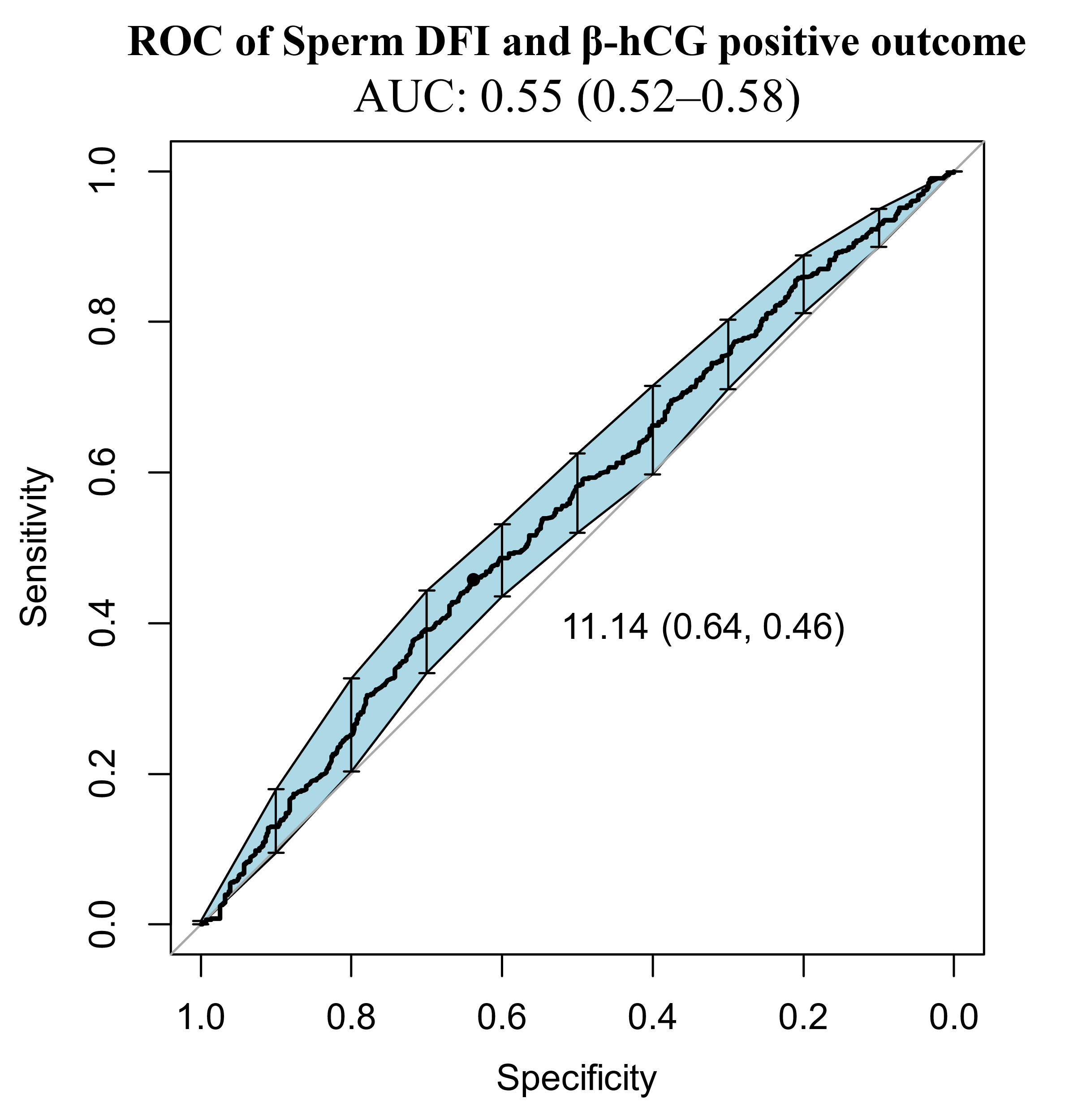 | |
| **Supplementary Fig. 6** Receiver operating characteristic (ROC) curves for sperm DFI and IVF outcomes. | |

| **Supplementary Table 1** Baseline characteristics of all participants in this study. | |
| --- | --- |
| **Characteristics** | **Overall** |
| N | 1258 |
| Duration of attempt to conceive, years | 3.0 [2.0, 6.0] |
| Male age, years | 33.0 [30.0, 36.0] |
| Female age, years | 31.0 [29.0, 34.0] |
| Male BMI, kg/m^2^ | 23.5 [21.5, 25.9] |
| Female BMI, kg/m^2^ | 21.9 [20.2, 23.9] |
| Sperm DNA fragmentation index, % | 12.8 [8.6, 19.0] |
| Semen volume, mL | 3.0 [2.1, 3.9] |
| Sperm concentration, 10^6^ / ml | 48.6 [30.2, 72.4] |
| Rapidly progressive motility, % | 34.0 [22.0, 45.0] |
| Slow or sluggish progressive motility, % | 19.0 [14.0, 22.8] |
| Anti-Mullerian hormone, ng/ml | 3.8 [2.5, 6.2] |
| Estradiol, pmol/L | 122.0 [87.0, 173.0] |
| Follicle-stimulating hormone, mIU/ml | 5.3 [4.5, 6.2] |
| Endometrial thickness on hCG trigger day, mm | 10.7 [9.5, 11.9] |
| Number of long down-regulation protocol | 862 (68.5) |
| Number of oocytes retrieved | 11.0 [8.0, 14.0] |
| Number of fertilized eggs | 9.0 [6.0, 11.0] |
| Number of oocytes cleaved | 9.0 [6.0, 11.0] |
| Number of embryos available on Day3 | 4.0 [2.0, 7.0] |
| Fertilization rate, % | 85.7 [71.4, 100.0] |
| Cleavage rate, % | 100.0 [100.0, 100.0] |
| D3 available embryos rate, % | 53.6 [33.3, 71.4] |
| β hCG positive outcome |  |
| Yes | 664 (52.8) |
| No | 594 (47.2) |
| Clinical pregnancy outcome |  |
| Yes | 646 (51.4) |
| No | 612 (48.6) |
| Live birth outcome |  |
| Yes | 549 (43.6) |
| No | 709 (56.4) |
| Note: Values are median (interquartile range) or number (%). | |

| **Supplementary Table 2** Distributions of the routine semen parameters and sperm DNA fragmentation index after Min-Max scaling. | | | | |
| --- | --- | --- | --- | --- |
| **Variables** | **25^th^ percentile** | **Median** | **Mean** | **75^th^ percentile** |
| Sperm DNA fragmentation index | 0.108 | 0.172 | 0.210 | 0.265 |
| Semen volume | 0.064 | 0.094 | 0.100 | 0.124 |
| Sperm concentration | 0.069 | 0.115 | 0.136 | 0.175 |
| Rapidly progressive motility | 0.313 | 0.493 | 0.486 | 0.657 |
| Slow or sluggish progressive motility | 0.240 | 0.340 | 0.329 | 0.415 |
|  | | | | |

| **Supplementary Table 3** P-values of overall and non-linear dose-response relationships of the sperm DFI and the studied routine semen parameters with IVF outcomes in adjustment of demographic characteristics and ovulation stimulation-related factors. | | |
| --- | --- | --- |
| **Variables** | ***P*-value for overall** | ***P*-value for non-linear** |
| **Live birth outcome** | | |
| Sperm DNA fragmentation index | **0.007** | 0.553 |
| Semen volume | 0.442 | 0.329 |
| Sperm concentration | 0.554 | 0.781 |
| Rapidly progressive motility | 0.106 | 0.221 |
| Slow or sluggish progressive motility | 0.283 | 0.119 |
| **Clinical pregnancy outcome** | | |
| Sperm DNA fragmentation index | **0.003** | 0.223 |
| Semen volume | 0.895 | 0.649 |
| Sperm concentration | 0.502 | 0.351 |
| Rapidly progressive motility | **0.025** | 0.114 |
| Slow or sluggish progressive motility | 0.202 | 0.110 |
| ***β*-hCG positive outcome** | | |
| Sperm DNA fragmentation index | **0.008** | 0.311 |
| Semen volume | 0.986 | 0.907 |
| Sperm concentration | 0.504 | 0.305 |
| Rapidly progressive motility | **0.040** | 0.199 |
| Slow or sluggish progressive motility | 0.161 | 0.062 |
| Notes: Bold indicates statistical significance. | | |

| **Supplementary Table 4** The crude and multi-variate adjusted odds ratios (95% CIs) of the IVF outcomes in relation to levels of the sperm DFI and the studied semen routine parameters. | | | |
| --- | --- | --- | --- |
| Variables | Crude model | Model 1 | Model 2 |
| ***Live birth outcome*** | | | |
| Sperm DFI | | | |
| Q1 | ref | ref | ref |
| Q2 | 0.741 (0.541, 1.013) | 0.764 (0.557, 1.047) | 0.755 (0.549, 1.038) |
| Q3 | **0.694 (0.506, 0.950)** | **0.710 (0.516, 0.976)** | **0.720 (0.522, 0.992)** |
| Q4 | **0.551 (0.400, 0.756)** | **0.580 (0.418, 0.803)** | **0.576 (0.414, 0.800)** |
| Semen volume | | | |
| Q1 | ref | ref | ref |
| Q2 | 1.278 (0.936, 1.748) | 1.262 (0.922, 1.730) | 1.285 (0.937, 1.765) |
| Q3 | 1.192 (0.868, 1.639) | 1.135 (0.822, 1.566) | 1.130 (0.818, 1.563) |
| Q4 | 1.151 (0.836, 1.584) | 1.113 (0.805, 1.539) | 1.135 (0.819, 1.573) |
| Sperm concentration | | | |
| Q1 | ref | ref | ref |
| Q2 | 1.329 (0.970, 1.825) | 1.365 (0.994, 1.876) | **1.384 (1.006, 1.906)** |
| Q3 | 1.088 (0.792, 1.496) | 1.096 (0.797, 1.508) | 1.109 (0.805, 1.529) |
| Q4 | 1.254 (0.914, 1.722) | 1.238 (0.901, 1.703) | 1.254 (0.911, 1.728) |
| Rapidly progressive motility | | | |
| Q1 | ref | ref | ref |
| Q2 | 1.289 (0.940, 1.770) | 1.252 (0.911, 1.722) | 1.264 (0.918, 1.741) |
| Q3 | **1.491 (1.090, 2.042)** | **1.417 (1.033, 1.948)** | **1.435 (1.044, 1.976)** |
| Q4 | 1.363 (0.993, 1.873) | 1.297 (0.942, 1.789) | 1.312 (0.951, 1.811) |
| Slow or sluggish progressive motility | | | |
| Q1 | ref | ref | ref |
| Q2 | **1.367 (1.015, 1.842)** | 1.331 (0.987, 1.797) | 1.335 (0.989, 1.806) |
| Q3 | 1.183 (0.843, 1.661) | 1.148 (0.815, 1.615) | 1.158 (0.820, 1.634) |
| Q4 | 1.093 (0.800, 1.495) | 1.074 (0.785, 1.471) | 1.078 (0.785, 1.479) |
| ***Clinical pregnancy outcome*** | | | |
| Sperm DFI | | | |
| Q1 | ref | ref | ref |
| Q2 | 0.739 (0.539, 1.013) | 0.740 (0.538, 1.016) | 0.738 (0.536, 1.016) |
| Q3 | **0.659 (0.480, 0.903)** | **0.649 (0.471, 0.894)** | **0.659 (0.478, 0.908)** |
| Q4 | **0.562 (0.409, 0.770)** | **0.563 (0.406, 0.778)** | **0.559 (0.403, 0.774)** |
| Semen volume | | | |
| Q1 | ref | ref | ref |
| Q2 | 1.139 (0.836, 1.551) | 1.128 (0.827, 1.540) | 1.142 (0.836, 1.562) |
| Q3 | 1.123 (0.820, 1.537) | 1.102 (0.802, 1.514) | 1.097 (0.798, 1.510) |
| Q4 | 1.192 (0.870, 1.634) | 1.193 (0.867, 1.642) | 1.212 (0.879, 1.673) |
| Sperm concentration | | | |
| Q1 | ref | ref | ref |
| Q2 | 1.323 (0.968, 1.811) | 1.333 (0.974, 1.827) | 1.354 (0.988, 1.858) |
| Q3 | 1.100 (0.805, 1.504) | 1.106 (0.809, 1.514) | 1.122 (0.819, 1.538) |
| Q4 | 1.250 (0.914, 1.710) | 1.237 (0.904, 1.695) | 1.257 (0.917, 1.724) |
| Rapidly progressive motility | | | |
| Q1 | ref | ref | ref |
| Q2 | 1.288 (0.944, 1.759) | 1.285 (0.940, 1.759) | 1.295 (0.946, 1.775) |
| Q3 | **1.558 (1.143, 2.128)** | **1.537 (1.123, 2.106)** | **1.557 (1.137, 2.137)** |
| Q4 | **1.368 (1.001, 1.871)** | 1.355 (0.988, 1.861) | 1.362 (0.992, 1.872) |
| Slow or sluggish progressive motility | | | |
| Q1 | ref | ref | ref |
| Q2 | 1.323 (0.985, 1.778) | 1.309 (0.974, 1.762) | 1.315 (0.977, 1.772) |
| Q3 | 1.359 (0.972, 1.904) | 1.325 (0.945, 1.860) | 1.337 (0.952, 1.881) |
| Q4 | 1.169 (0.860, 1.591) | 1.166 (0.856, 1.589) | 1.167 (0.855, 1.593) |
| ***β-hCG positive outcome*** | | | |
| Sperm DFI | | | |
| Q1 | ref | ref | ref |
| Q2 | 0.819 (0.596, 1.122) | 0.817 (0.594, 1.123) | 0.816 (0.592, 1.124) |
| Q3 | **0.693 (0.505, 0.950)** | **0.688 (0.499, 0.946)** | **0.699 (0.506, 0.962)** |
| Q4 | **0.607 (0.442, 0.831)** | **0.611 (0.441, 0.843)** | **0.607 (0.438, 0.841)** |
| Semen volume | | | |
| Q1 | ref | ref | ref |
| Q2 | 1.095 (0.804, 1.491) | 1.090 (0.799, 1.489) | 1.103 (0.807, 1.508) |
| Q3 | 1.068 (0.780, 1.462) | 1.052 (0.766, 1.445) | 1.045 (0.759, 1.437) |
| Q4 | 1.212 (0.884, 1.662) | 1.215 (0.883, 1.674) | 1.238 (0.897, 1.709) |
| Sperm concentration | | | |
| Q1 | ref | ref | ref |
| Q2 | 1.358 (0.993, 1.860) | 1.369 (0.999, 1.876) | **1.391 (1.014, 1.911)** |
| Q3 | 1.086 (0.795, 1.485) | 1.090 (0.797, 1.491) | 1.103 (0.805, 1.512) |
| Q4 | 1.266 (0.926, 1.733) | 1.251 (0.914, 1.714) | 1.269 (0.926, 1.740) |
| Rapidly progressive motility | | | |
| Q1 | ref | ref | ref |
| Q2 | 1.277 (0.936, 1.744) | 1.274 (0.932, 1.743) | 1.286 (0.940, 1.762) |
| Q3 | **1.467 (1.076, 2.003)** | **1.447 (1.058, 1.982)** | **1.463 (1.068, 2.007)** |
| Q4 | **1.376 (1.007, 1.883)** | 1.360 (0.992, 1.867) | 1.367 (0.996, 1.879) |
| Slow or sluggish progressive motility | | | |
| Q1 | ref | ref | ref |
| Q2 | 1.283 (0.955, 1.724) | 1.270 (0.945, 1.710) | 1.273 (0.946, 1.716) |
| Q3 | 1.280 (0.915, 1.793) | 1.246 (0.889, 1.750) | 1.262 (0.898, 1.776) |
| Q4 | 1.091 (0.802, 1.484) | 1.082 (0.795, 1.474) | 1.077 (0.790, 1.470) |
| Abbreviations: DFI, DNA fragmentation index; OR, odds ratio; CI, confidence interval; BMI, body mass index; AMH, Anti-Mullerian hormone; E2, Estradiol; FSH, Follicle-stimulating hormone.  Notes: Model 1 was adjusted for duration of attempt to conceive, female age, male age, female BMI, and male BMI. Model 2 was further adjusted for controlled ovulation stimulation protocols, AMH, E2, FSH, endometrial thickness, and numbers of oocytes retrieved.  Bold indicates statistical significance. | | | |

| **Supplementary Table 5** Mediation analysis with IVF outcomes in association with clusters and fertilization rate. | | | | |
| --- | --- | --- | --- | --- |
|  | **Total effect**  **(95%CI)** | **ACME**  **(95% CI)** | **ADE**  **(95% CI)** | **Mediation proportion**  **(95% CI)** |
| **Clusters → Fertilization rate → Live birth outcome** | | | | |
| Cluster 1 | ref | ref | ref | ref |
| Cluster 2 | 0.03  (-0.07 ~ 0.12) | -0.002  (-0.01 ~ 0.01) | 0.03  (-0.07 ~ 0.12) | -0.2%  (-1.5% ~ 1.0%) |
| Cluster 3 | -0.05 (-0.15 ~ 0.06) | **-0.02 (-0.04 ~ 0.00)** | -0.03  (-0.13 ~ 0.07) | 24.8%  (21.2% ~ 27.9%) |
| Cluster 4 | -0.12  (-0.26 ~ 0.04) | **-0.06 (-0.12 ~ -0.01)** | -0.06  (-0.21 ~ 0.10) | 44.1%  (41.0% ~ 48.1%) |
| **Clusters → Fertilization rate → Clinical pregnancy outcome** | | | | |
| Cluster 1 | ref | ref | ref | ref |
| Cluster 2 | 0.07  (-0.03 ~ 0.16) | -0.002  (-0.02 ~ 0.01) | 0.07  (-0.02 ~ 0.16) | -1.7%  (-2.6% ~ -1.1%) |
| Cluster 3 | -0.05  (-0.15 ~ 0.06) | **-0.02**  **(-0.05 ~ 0.00)** | -0.02  (-0.12 ~ 0.07) | 27.7%  (24.3% ~ 31.2%) |
| Cluster 4 | -0.13  (-0.28 ~ 0.02) | **-0.06**  **(-0.12 ~ -0.01)** | -0.07  (-0.23 ~ 0.08) | 42.6%  (41.6% ~ 46.2%) |
| **Clusters → Fertilization rate → *β*-hCG positive outcome** | | | | |
| Cluster 1 | ref | ref | ref | ref |
| Cluster 2 | 0.05  (-0.05 ~ 0.14) | -0.002  (-0.02 ~ 0.01) | 0.05  (-0.04 ~ 0.14) | -1.1%  (-2.3% ~ 0.4%) |
| Cluster 3 | -0.04  (-0.14 ~ 0.07) | **-0.02**  **(-0.05 ~ 0.00)** | -0.02  (-0.12 ~ 0.08) | 27.4%  (23.7% ~ 32.5%) |
| Cluster 4 | -0.14  (-0.29 ~ 0.01) | **-0.06**  **(-0.11 ~ -0.01)** | -0.09  (-0.24 ~ 0.06) | 36.5%  (35.8% ~ 39.4%) |
| Abbreviations: ACME, average causal mediation effects; ADE, average direct effects; CI, confidence interval.  Bold demonstrates statistical significance. | | | | |
